# An empirical Bayes model for gene expression and methylation profiles in antiestrogen resistant breast cancer

**DOI:** 10.1186/1755-8794-3-55

**Published:** 2010-11-25

**Authors:** Jaesik Jeong, Lang Li, Yunlong Liu, Kenneth P Nephew, Tim Hui-Ming Huang, Changyu Shen

**Affiliations:** 1 Department of Medicine/Division of Biostatistics, Indiana University, Indianapolis, IN, USA; 2 Department of Medical and Molecular Genetics, Indiana University, Indianapolis, IN, USA; 3 Department of Medical Science/Simon Cancer Center, Indiana University, Bloomington, IN, USA; 4 Department of Molecular Virology, Immunology and Medical Genetics/Human Cancer Genetics, The Ohio State University, Columbus, OH, USA

## Abstract

**Background:**

The nuclear transcription factor estrogen receptor alpha (ER-alpha) is the target of several antiestrogen therapeutic agents for breast cancer. However, many ER-alpha positive patients do not respond to these treatments from the beginning, or stop responding after being treated for a period of time. Because of the association of gene transcription alteration and drug resistance and the emerging evidence on the role of DNA methylation on transcription regulation, understanding of these relationships can facilitate development of approaches to re-sensitize breast cancer cells to treatment by restoring DNA methylation patterns.

**Methods:**

We constructed a hierarchical empirical Bayes model to investigate the simultaneous change of gene expression and promoter DNA methylation profiles among wild type (WT) and OHT/ICI resistant MCF7 breast cancer cell lines.

**Results:**

We found that compared with the WT cell lines, almost all of the genes in OHT or ICI resistant cell lines either do not show methylation change or hypomethylated. Moreover, the correlations between gene expression and methylation are quite heterogeneous across genes, suggesting the involvement of other factors in regulating transcription. Analysis of our results in combination with H3K4me2 data on OHT resistant cell lines suggests a clear interplay between DNA methylation and H3K4me2 in the regulation of gene expression. For hypomethylated genes with alteration of gene expression, most (~80%) are up-regulated, consistent with current view on the relationship between promoter methylation and gene expression.

**Conclusions:**

We developed an empirical Bayes model to study the association between DNA methylation in the promoter region and gene expression. Our approach generates both global (across all genes) and local (individual gene) views of the interplay. It provides important insight on future effort to develop therapeutic agent to re-sensitize breast cancer cells to treatment.

## Background

The term epigenetics in general refers to heritable pattern of gene expression that is mechanistically regulated through processes other than alteration in the primary DNA sequences [1,2]. Epigenetics has implications in both our understanding of gene regulation in complex organisms such as mammals and clinical investigation on various diseases such as cancer [3,4]. It is now clear that epigenetic events can occur at both the DNA level (i.e. DNA methylation) and chromatic level (i.e. histone modifications), resulting in an intricate process of interactions that ultimately lead to the alteration of gene expression [5-7].

DNA methylation is a process that adds a methyl group to the cytosine ring via a co-valent bond, using S-adenosyl-methionine as the methyl donor and DNA methyltransferases (DNMTs) as the catalytic enzyme [5]. In mammals, DNA methylation is mostly common on cytosines that precede a guanosine (the CpG dinucleotide). Two features characterize the distribution of the CpG dinucleotides in the genome. First, the overall frequency of the CpG dinucleotides is substantially less than one would expect from probabilistic calculations, which is likely due to a depletion process induced by methylation over time [8]. Second, the distribution of CpG dinucleotides in the genome is highly asymmetric with a high concentration of DNA segments 200bp to several kb in length called "CpG islands", residing in the promoter region and first exon for approximately 60% of genes [6]. A striking feature that distinguishes CpG islands from CpG dinucleotides is that under normal conditions, CpG islands generally lack DNA methylation, whereas CpG dinucleotides are typically methylated (i.e. 80%) [2]. While the relationship between CpG island methylation and gene silencing is well established, the mechanisms underlying this phenomena are less clear but thought to include physical blocking of transcription factor binding [9,10] and/or recruitment of transcriptional repressors to the methylated sites [11].

A more complete understanding of the DNA methylation in carcinogenesis is beginning to emerge. A general observation is that the level and pattern of DNA methylation in cancer cells is the opposite of their normal counterparts. The cancer methylome is characterized by global hypomethylation of DNA, which is linked primarily to repeated DNA sequences becoming hypomethylated. Hypomethylation may contribute to carcinogenesis by promoting tumor formation or progression in a number of possible ways, including affecting transposable element activation, DNA/chromosomal rearrangements, tumor suppressor gene or oncogene copy number, and/or altered chromosome conformation. In contrast to normal cells, increased methylation of CpG islands is a common occurrence in cancer, and is associated with epigenetic silencing during all phases of the cancer process, including tumor initiation, progression and drug resistance. Aberrant CpG island methylation is associated with silencing of genes involved in control of the cell cycle, apoptosis and drug sensitivity, as well as tumor suppressor genes.

Although the above phenomena are well documented in all cancers and recognized as playing an important role in almost every aspect of carcinogenesis, the mechanistic nature of the relationship between methylation and regulation of gene expression remains incompletely understood, including the heterogeneity of the relationship among genes, the interaction of methylation at different sites and the involvement of other epigenetic events.

In the clinical setting, a critical issue for cancer treatment is acquired drug resistance, where patients initially respond to chemotherapy but cease to respond after repeated exposure to the same drug. Potentially, epigenetic alterations, such as DNA methylation, are likely to play an important role in acquired drug resistance, as suggested by several studies [12-15], though much work is yet to be done to gain a clear insight into this phenomenon. Based on our experience in studies of hormone-therapy resistance in breast cancer, antiestrogen resistance is accompanied by dramatic alterations in the expression level of many genes, and alteration of DNA methylation may be one of the causes.

In this article, we focused on understanding the association between CpG island methylation and gene expression in breast cancer. In particular, we attempted to gain a better understanding of differences in DNA methylation and gene expression between hormone-therapy-sensitive and -resistant cell lines. We considered two breast cancer cell lines that are resistant to tamoxifen and fulvestrant, respectively. These are two clinically important therapeutic agents that target estrogen receptor alpha (ER-alpha), a nuclear receptor that primarily mediates genomic regulation of gene transcription and non-genomic activation of various kinase pathways [16]. It is well known that ER-alpha is a key protein implicated in the majority of breast cancers. Although both tamoxifen and fulvestrant are antagonists of ER-alpha, their mechanisms of action differ markedly [17]. Tamoxifen functions as a competitive agent of E2 (the ligand that stimulates ER-alpha), blocking E2 binding to ER-alpha. In spite of this antagonistic action, tamoxifen-bound ER-alpha is capable of regulating gene transcription through genomic/non-genomic actions. On the other hand, fulvestrant directly inhibits the process through which ER-alpha executes genomic regulation function, rapidly inducing cytoplasm aggregation and ER-alpha degradation [17].

Based on their different mechanisms of action, the transition to a resistant state by constant exposure to these agents likely involves both similar and distinct molecular alterations. The aim of this study is to identify at both the individual gene as well as genome level the regulation status in both DNA methylation and gene expression by comparing drug-resistant cell lines to drug-sensitive cell lines. This study provides important insight on the search of potential targets for epigenetic therapy to re-sensitize tumor cells to hormone or chemo-therapy. Toward this goal, we developed an empirical Bayes statistical model to integrate gene expression and DNA methylation data. Advantages of such a model include (i) consideration of probe-probe variation, (ii) easily interpretable confidence of the detections and (iii) straight forward false discovery rate (FDR) control/estimate [18,19].

## Methods

### Experiment

The Human Genome U133A 2.0 Array was used for gene expression analysis. We restricted our analysis to probes with at least two "present" calls among four replicates. Differential methylation hybridization (DMH) was done using customized 60-mer oligonucleotide microarrays, which contain ~44,000 CpG-rich fragments from ~12,000 promoters of defined genes [20]. Microarray Analysis Suite (MAS) version 5.0 was used for preprocessing. Experimental details were described in [20]. The data discussed in this paper have been deposited in NCBI's Gene Expression Omnibus http://www.ncbi.nlm.nih.gov/geo/ and are accessible through GEO Series accession number (GSE5840 for gene expression and GSE25519 for methylation).

### Data

**Figure 1:**
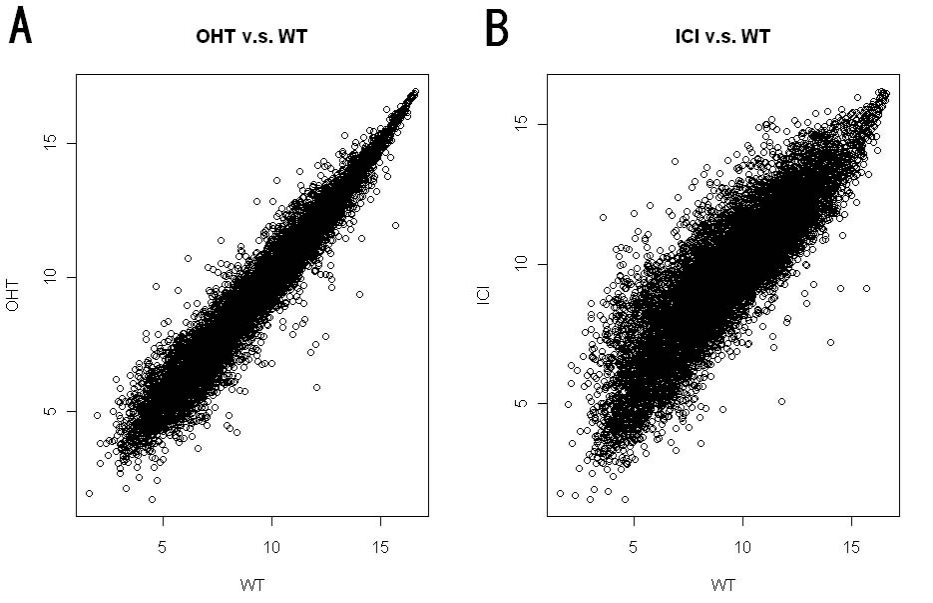
**Gene expression data plot**. Gene expression data (average over samples within each group): OHT v.s. WT and ICI v.s. WT at log2 scale.

**Figure 2:**
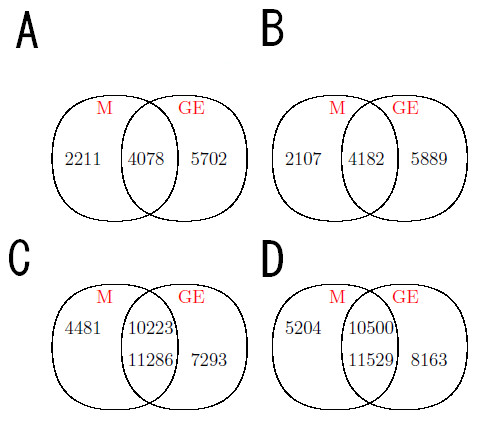
**Number of probes and genes**. Summary of the number of genes and probes included in our statistical analysis after the preprocessing; Panel A and C: numbers of genes (A) and probes (C) for OHT v.s. WT; Panel B and D: number of genes (B) and probes (D) for ICI v.s. WT. Numbers in the intersection area represent the genes or probes included in the statistical analysis. GE: gene expression data; M: DNA methylation data.

We compared gene expression and DNA methylation in tamoxifen-(OHT; 4-hydroxytamoxifen) and fulvestrant (ICI 182,780)-resistant MCF7 breast cancer cell lines to the parental, wild type (WT) MCF7 cell line [20]. There were four replicates for gene expression data and no replicate for the methylation data. The structure for the gene expression data is given in Additional file 1. We restricted our analysis to probes with at least two "present" calls among four replicates in gene expression and our focus in methylation is on promoter region probes. Then, for WT v.s. OHT, the gene expression data have a total of 11286 probes that cover 4078 genes, while methylation data have a total of 10223 probes and 4078 genes. For WT v.s. ICI, there are 11529 probes for gene expression and 10500 methylation probes. In addition, there are 4182 genes in common. The gene expression data plots (average over replicates within each group) are given in Figure 1 as an illustration. We also summarized the number of genes and probes included in our statistical analysis after the preprocessing (Figure 2). Probe intensities are log2 transformed before analysis for both gene expression and methylation data.

### The Model

We consider an empirical Bayes model to correlate alteration of gene expression and DNA methylation in WT and drug-resistant cell lines. The marginal model for gene expression (*G_ijkl_*) is given by:

(1) $$\begin{array}{c} G_{ijkl}=\mu_{il}+b_{ij}+\epsilon_{ijkl},\\ i=1,\;\cdots ,\;I,\;j=1,\;\cdots ,\;J_{i},\;k=1,\;\cdots ,\;K,\;l\;=1,2 \end{array}$$

where *i *indexes gene, *j *indexes probe in a gene, *k *indexes replicates, and *l *denotes WT (*l *= 1) and resistant group (*l *= 2). Therefore, μ*_il _*is the average expression level for gene *i *in cell line *l*, *b_ij _*is the added effect of probe *j *for each gene *i*, and ε*_ijkl _*is the error term. We consider the following distributions for each component in the model:

(2) $$b_{ij}\sim N(0,\sigma^{2}),\;\epsilon_{ijkl}\sim N(0,\delta^{2}),\;{\left(\mu_{i1},\mu_{i2}\right)}^{t}\sim N({\left(\mu_{1},\mu_{2}\right)}^{t},\Sigma_{1}).$$

Marginal model for methylation (*M_ihl_*) is given in a similar fashion:

(3) $$M_{ihl}=\eta_{i}{}_{l}+a_{ih}+d_{ihl},\;i=1,\;\cdots ,\;I,\;h=1,\;\cdots ,\;H_{i},\;l=1,2$$

Similarly, *η_il _*is gene effect in each group, *a_ij _*is the probe effect in each gene, and *d_ihl _*is the error term. However, no replicate is here in contrast to GE data. Again, we assume the same distributions for components in Eq. (3):

(4) $$a_{ih}\sim N(0,\omega^{2}),\;d_{ihl}\sim N(0,\tau^{2}),\;{\left(\eta_{i1},\eta_{i2}\right)}^{t}\sim N({\left(\eta_{1},\eta_{2}\right)}^{t},\Sigma_{2}).$$

To integrate both marginal models into an unified model, we assume

(5) $${\left(\mu_{i1},\mu_{i2},\eta_{i1},\eta_{i2}\right)}^{t}\sim N(\mu ,\Sigma )$$

where $\Sigma =\left(\begin{array}{cc} \sum_{1} & \sum^{*}\\ \sum^{*} & \sum_{2} \end{array}\right)$.

Models (1) - (5) can be represented in a simple linear model format [21,22]:

(6) $$D_{i}=\Delta_{i}\beta_{i}+\epsilon_{i},\;i=1,\;\cdots ,\;I$$

where $\beta_{i}={\left(\mu_{i1},\mu_{i2},\eta_{i1},\eta_{i2},b_{1},\cdots ,b_{J_{i}},a_{1},\cdots ,a_{H_{i}}\right)}^{t}$, *D*_*i *_= (*G*_*i*_^*t*^, *M*_*i*_^*t*^)^*t*^. *G*_*i *_and *M*_*i *_are gene expression and methylation data for gene *i*, respectively. The distribution assumption for each component in the unified model (Eq. (6)) is:

(7) $$\beta_{i}\sim N(\mu_{{}_{i}}^{*},\Sigma_{pi}),\epsilon_{i}\sim N(0,\Sigma_{e_{i}})$$

where $\mu_{i}^{*}={\left(\mu ,0\right)}^{t}$,

$$\begin{array}{cc} \sum_{pi}=\left(\begin{array}{ccc} \sum & 0 & 0\\ 0 & \sigma^{2}I_{J_{i}} & 0\\ 0 & 0 & \omega^{2}I_{H_{i}} \end{array}\right), & \sum_{e_{i}}=\left(\begin{array}{cc} \delta^{2}I_{N_{i}} & 0\\ 0 & \tau^{2}I_{M_{i}} \end{array}\right). \end{array}$$

Here *N_i _*= 2*K *× *J*_*i *_and *M_i _*= 2*H_i_*. As we can see, our model has hierarchical structure for each gene *i*:

(8) $$\begin{array}{cc} D_{i}|\beta_{i}\sim N(\Delta_{i}\beta_{i},\Sigma_{e_{i}}), & \text{prior}\;\text{of}\beta_{i}\sim N(\mu_{i}^{*},\Sigma_{pi}) \end{array}$$

Thus, posterior distribution of β*_i_*|*D_i _*follows *N*(K, K _*_) where $K=K^{*}(\Delta_{i}{}^{T}\Sigma_{e_{i}}{}^{-1}D_{i}+\Sigma_{pi}{}^{-1}\mu_{i}^{*})$ and $K^{*}={\left(\Delta_{i}{}^{T}\Sigma_{e_{i}}{}^{-1}\Delta_{i}+\Sigma_{pi}{}^{-1}\right)}^{-1}$. Inference is based on the posterior distribution above.

More details about statistical modeling are provided in Additional file 2.

### Estimation

Expectation-Maximization (EM) algorithm [23,24] is widely used to obtain maximum likelihood estimates when there are unobserved variables. Basically, EM algorithm consists of two iterative steps: Expectation and Maximization. In the E-step, expectation of complete-data log likelihood conditional on data and current value of parameters is calculated. In the M-step, parameters are updated by the value that maximizes the expectation from E-step. Here we briefly describe EM algorithm applied to our case. More details about E-step and M-step are given in Additional file 2.

### E-step

For iteration *k*, we obtain *E*(*β_i_*|*D_i_*, *θ *^(*k *- 1)^) and *Cov*(*β_i_*|*D_i_*, *θ *^(*k *- 1)^) that allows us to compute

$$Q(\theta ;\theta^{\left(k-1\right)})\equiv E[\log L_{c}(\theta )|D_{i},\theta^{\left(k-1\right)}].$$

Here $\log L_{c}(\theta )=\sum_{i=1}^{I}\{\log[D_{i},\beta_{i}|\theta ]\}$ is complete-data log likelihood function where *θ *is the parameter vector.

### M-step

In this step, we update *θ *by values that maximize the target function, *Q*(*θ*; *θ *^(*k *- 1)^) given in the E-step.

### Inference on Relationship between Gene Expression and Methylation

Inference on relationship between gene expression and methylation can be made by using posterior distribution of (*μ*_*i*1_, *μ*_*i*2_, *η*_*i*1_, *η*_*i*2_)^*t *^conditional on data and parameter estimates, where *μ*_*i*1 _and *η*_*i*1 _are mean parameters of gene expression and methylation in WT and *μ*_*i*2 _and *η*_*i*2 _are mean parameters of gene expression and methylation in the resistant cell line. Since we are interested in the correlation of the differentiation of gene expression and DNA methylation, we focus on the posterior distribution of

(9) $${\left(\mu_{GE_{i}},\eta_{M_{i}}\right)}^{t}={\left(\mu_{i2}-\mu_{i1},\eta_{i2}-\eta_{i1}\right)}^{t},$$

which can be easily calculated through a linear transformation of (*μ*_*i*1_, *μ*_*i*2_, *η*_*i*1_, *η*_*i*2_)*^t^*.

To characterize the correlation of gene expression and DNA methylation for each gene, we first divide the two-dimensional sample space of $\left(\mu_{GE_{i}},\eta_{M_{i}}\right)$ into nine categories by applying two thresholds to each of the $\mu_{GE_{i}}$ and $\eta_{M_{i}}$ dimensions. The nine categories represent the combination of three levels of alteration in gene expression and DNA methylation: up-regulation, no change, down-regulation. For instance, the north-east region will be "up-regulation in both expression and DNA methylation". The thresholds are chosen to be ± C _* _σ, where σ is the standard deviation of the posterior mean of $\mu_{GE_{i}}$ or $\eta_{M_{i}}$ across all genes. In our application, we chose C = 1.5. We then calculate for each gene the posterior probability of each of the nine regions, which characterizes the correlation of gene expression and DNA methylation for each gene in a probabilistic manner. Based on these probabilities, we will assign each gene to one of the nine categories. See result section for details.

## Results and Discussion

### Association between Gene Expression and Methylation Status

The main output from our model is a joint posterior distribution of the difference of expression and methylation levels between drug-resistant cell lines and WT for each gene. Such a distribution provides us with a probabilistic measure on the strength of the association for each gene. On the other hand, the center (or the mean) of the prior distribution of the difference of expression and methylation provides us with a global view of the association across genes. In the following discussion, up and down regulation is always in reference to the WT.

**Figure 3:**
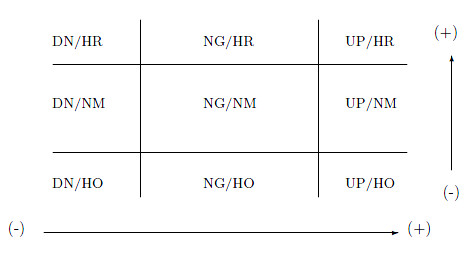
**Nine categories for gene assignment**. 9 categories (X-axis: gene expression; Y-axis: methylation status); NG, DN, and UP stand for no change, down regulation, and up regulation in gene expression. NM, HO, and HR stand for no change in methylation, hypomethylation, and hypermethylation.

**Figure 4:**
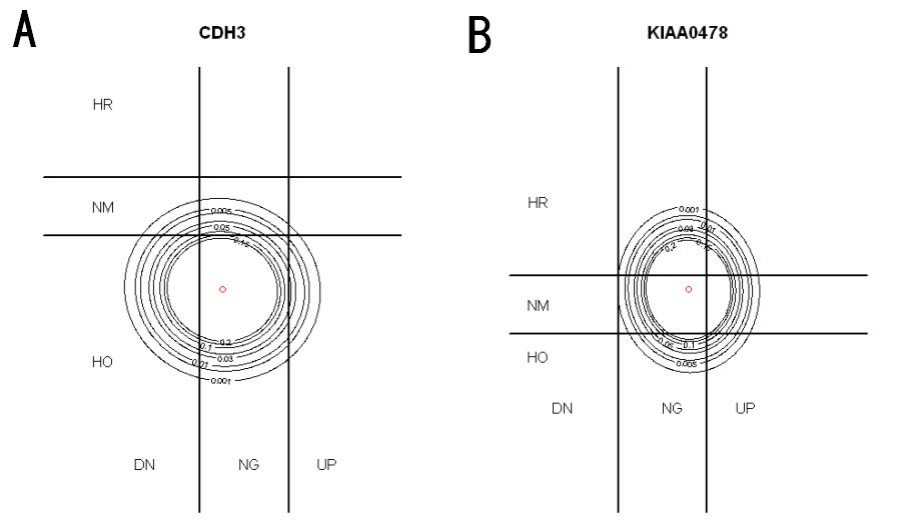
**Example of gene assignment**. Contour plot of posterior distribution of two genes (Left: *CDH3*; Right: *KIAA0478*).

To facilitate the understanding of the association between gene expression and DNA methylation at the individual gene level, we used four thresholds (two for gene expression and two for methylation) to categorize each gene into nine categories (Figure 3). In the figure, NG, UP, and DN stand for no change, up regulation, and down regulation in gene expression. Similarly, NM, HO, and HR stand for no change in methylation, hypomethylation and hypermethylation. For example, the upper-left cell is the category for down regulation of gene expression and hypermethylation. The thresholds are determined based on the posterior distribution (see Method section). Given the thresholds, one can calculate posterior probability that each gene falls into one category. We then assign each gene to one of the nine categories based on these probabilities. We consider two ways of categorization. In the first method, a gene is assigned to the category with the maximum probability. The limitation of this method is that when the probability mass is evenly distributed among the several categories, the maximum is not a substantially dominating number, which still entails a lot of uncertainty. Therefore, in the second method, we apply a threshold to the maximum probability for gene assignment. Genes with maximum probability below the threshold are not assigned to any category. In the context of FDR, applying a cutoff value to the posterior probability means that the false discovery rate is controlled at α = *D*(*κ*) = *K*, where $K=\sum_{i}I_{\left[P_{i}{}^{s}\geq \kappa \right]}$ i.e., the number of genes whose posterior pass the threshold *k *and *D*(*κ*) is the summation of one minus the posterior probabilities of these genes:

(10) $$D(\kappa )=\sum_{i}\{1-P_{i}^{s}\}I_{\left[P_{i}{}^{s}\geq \kappa \right]}$$

where *I *is the indicator function and *P_i_^s ^*is the posterior probability of gene *i *belonging subcategory *s *[19]. We illustrate these definitions through two examples. Figure 4 is the contour plots of the posterior distributions of two genes (*CDH3, KIAA0478*) from the OHT vs. WT comparison superseded on nine categories. It is well known that gene *CDH3*, which acts as a tumor suppressor gene, is hypomethylated in breast cancer [6]. This is visually confirmed by Figure 4 by observing that most of the probability mass is concentrated on the region of no expression change but reduced methylation. It can be shown that the probability of this category is 0.8. On the other hand, *KIAA0478 *concentrates on the region of no expression and methylation change with a probability of 0.63. To characterize all genes assigned into one of the nine categories, we apply three assignment rules: maximum probability, maximum probability over 0.6 and maximum probability over 0.7. Table 1 and 2 summarize the results for OHT v.s. WT and ICI v.s. WT, respectively. The gene lists obtained from using three different cutoff values are given in Additional file 3.

**Table 1:** Gene assignment to nine category

	Gene expression		
**Methylation**	**DOWN**	**No change**	**UP**

HYPER	0/0/0	1/0/0	0/0/0
No change	101/45/17	2284/1382/786	176/63/35
HYPO	48/11/6	1331/744/496	137/46/24

Three values in each cell present the number of genes belonging to each category using three different assignment rules: maximum probability, maximum probability over 0.6, and maximum probability over 0.7.(OHT v.s. WT)

**Table 2:** Gene assignment to nine category

	Gene expression		
**Methylation**	**DOWN**	**No change**	**UP**

HYPER	0/0/0	1/1/0	0/0/0
No change	86/20/9	2309/1518/922	237/114/64
HYPO	50/12/7	1350/811/526	149/61/40

Three values in each cell present the number of genes belonging to each category using three different assignment rules: maximum probability, maximum probability over 0.6, and maximum probability over 0.7.(ICI v.s. WT)

Not surprisingly, the category NG/NM contains most of the genes. The other feature is that very few genes are hypermethylated, similar to what was observed in the original report of the experiments [20]. While the reason for this is not clear, one possibility is that hypomethylation and up regulation of the corresponding gene(s) may provide the drug-resistant cells with a survival and growth advantage. Among those hypomethylated genes with expression alteration, the majority are up-regulated, consistent with what is known regarding promoter methylation and gene expression. The hypomethylated, down-regulated genes suggest other mechanisms are involved in regulating expression in addition to DNA methylation, such as the repressive histone methylation [25-31].

**Figure 5:**
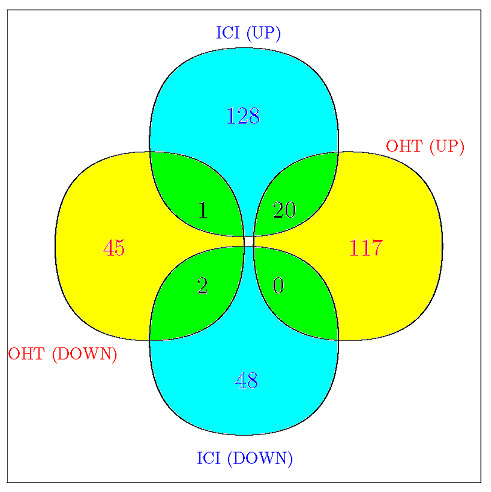
**Venn diagram presenting the number of genes in each Gene expression status**. Venn diagram of UP/DOWN genes between OHT and ICI using maximum probability; UP and DOWN stand for up and down regulation in gene expression, respectively. Genes are restricted to hypomethylated ones.

**Figure 6:**
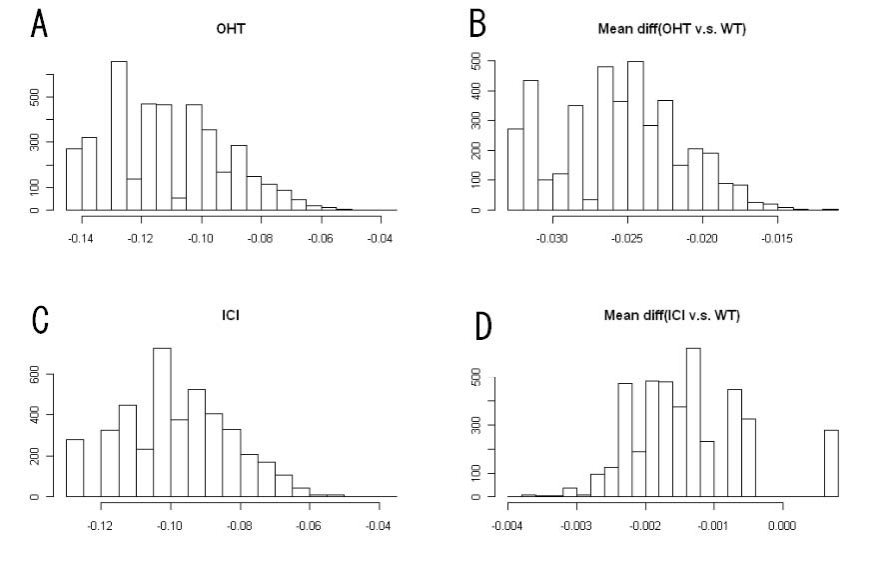
**Histogram of gene-specific correlations**. Panel A: histogram of correlations between gene expression and methylation in OHT; Panel B: histogram of correlations between mean difference gene expression and methylation (OHT v.s. WT); Panel C: histogram of correlations between gene expression and methylation in ICI; Panel D: histogram of correlations between mean difference gene expression and methylation (ICI v.s. WT).

With hypomethylated genes only, the Venn diagram for the overlap of genes with expression alteration between the two drug-resistant cell lines is shown in Figure 5 (based on maximum probability criterion). It can be seen that the overall overlap is small, suggesting distinct sets of hypomethylated genes between OHT and ICI resistant breast cancer cells. The number of overlapping genes that are up-regulated in both cell lines (i.e., 20) is disproportionally higher than the overlapping genes for down regulation patterns (i.e., 2), suggesting that the association of DNA hypomethylation and up regulation in both cell lines may share a common gene set, at least more common than the other association patterns. More details about overlaps for different cutoffs are given in Additional file 4. At the global level, the Pearson correlation coefficients (across genes) between DNA methylation and gene expression are -0.17 for WT, -0.19 for OHT and -0.16 for ICI. Hence, there seems to be some relationship, in general, between DNA methylation and gene expression, and although the magnitude is not strong, this observation is consistent with the general understanding that increased promoter DNA methylation correlates with a lower level of gene activity. From another angle, if we examine the correlation between methylation and gene expression alterations (i.e., $corr\;({\hat{\mu }}_{i2}-{\hat{\mu }}_{i1},{\hat{\eta }}_{i2}-{\hat{\eta }}_{i1})$ in Eq. (9)) when comparing OHT/ICI to WT, the correlation coefficients across genes are -0.04 for OHT and 0.01 for ICI, implying methylation and gene regulation are not strongly associated in this model system, and this relationship may be highly gene-specific. The local level (gene-specific) view is given in the following histogram (Figure 6). Panels A and C present the histograms of correlations between gene expression and methylation in OHT and ICI. Panels B and D present distribution of correlations between gene expression mean difference and methylation mean difference obtained from OHT v.s. WT and ICI v.s. WT, respectively. Panels A and C show the distribution of correlations between gene expression and methylation ranging from -0.15 to 0, implying heterogeneity in gene-specific correlations.

### Histone Methylation

It is well known that the interplay of histone modification and DNA methylation affects the transcriptional regulation [25-29]. To examine the involvement of histone methylation in the association of alterations of DNA methylation and gene expression, we analyzed some in-house histone methylation data. The data were generated by chromatin-immunoprecipitation and high-throughput sequencing (ChIP-seq). The experimental protocol followed the same line of procedure reported previously [32,33]. We will focus our discussion on the dimethylation on lysine residue 4 on H3 (H3K4me2) on OHT MCF7 cell lines. Our data include 26443 genes with two replicates. We first compared H3K4me2 levels in OHT between genes with DNA hypomethylation and those without alteration in DNA methylation, i.e. the third row versus the second row in Table 1 (maximum probability rule is used to assign genes to a category). The fold change is 1.10 (95% CI: 1.00-1.20). Therefore, DNA hypomethylation is associated with enhanced H3K4me2 in this setting. This observation is intuitively appealing as both DNA hypomethylation and H3K4me2 were found to be related to gene activation [27,28,30] and [31].

We next compared the H3K4me2 levels for (i) genes in the UP/HO category versus those in the DN/HO category; (ii) UP/NM category versus DN/NM category (Table 1). The fold changes are 1.56 (95% CI 0.89-2.72) and 1.24 (95% CI: 0.82-1.88), respectively. Therefore, consistent with previous findings [27,28,30,31], our results show that H3K4me2 is likely to be associated with transcriptional activation. Moreover, there seems to be a higher level of H3K4me2 change in genes with DNA hypomethylation than those without DNA methylation change, suggesting an interaction between H3K4me2 and DNA methylation in regulating gene expression.

### Gene Ontology Analysis

Although it is likely that other genetic or epigenetic activities in addition to DNA methylation are involved in the regulation of genes, our finding is consistent with the general observation that hypomethylation leads to up regulation. Furthermore, we conducted a gene ontology analysis on these genes and the results are shown in Table 3. Several functional categories are over-represented in both cell lines, such as cell death, connective tissue development and function and cellular development. On the other hand, distinct functional categories are associated with each cell line. Taken together, these observations suggest while common mechanisms for switching of genes through DNA demethylation at promoter regions are shared by OHT- and ICI-resistant cells, unique processes are also associated with development of acquired resistance to the distinctly different antiestrogens. These distinct functional and molecular changes associated with the acquisition of resistance to the two different classes of antiestrogens include signaling and growth regulatory processes.

**Table 3:** Gene Ontology Analysis

OHT	ICI
Cell Death	Cell Death
Connective Tissue Development & Function	Connective Tissue Development & Function
Cellular Development	Cellular Development
Cellular Compromise	Immune Cell Trafficking
Infectious Disease	Nervous System Development & Function
Post-Translational Modification	Organismal Development
Carbohydrate Metabolism	Cellular Movement
Cell-To-Cell Signaling and Interaction	Hematological System Development & Function
Cellular Assembly and Organization	Cellular Function and Maintenance
Cell Morphology	Hematological Disease

This table includes top 10 over-represented functional categories for genes with hypomethylation and up regulation of gene expression with maximum probability

## Conclusions

In this article, we developed an empirical Bayes model to study the association between altered DNA methylation in the promoter region and gene expression by comparing WT with OHT and ICI resistant MCF7 breast cancer cell lines. Our statistical model incorporates various sources of variations that generate probabilistic characterization of such an association. The model structure also allows a natural incorporation of other epigenetic processes to investigate their regulatory roles in acquired antiestrogen resistance.

Our models are characterized by a hierarchical structure that has been shown to be more efficient and stable than analysis of individual gene separately [34]. It also allows one to estimate the correlation between gene expression and DNA methylation at the level of individual genes. However, our models induce a marginally positive correlation between probes of the same gene, which might not hold for all genes and all microarray platforms. A small simulation study (data not shown) suggests that the inference on gene level quantity μ*_il _*and *η_il _*is relatively robust when probes are actually negatively correlated. Finally, given the complexity of our model it is not possible to use standard diagnostic tools to check model assumptions. Nevertheless, it is still possible to examine posterior quantities of latent variable that is conditional on the data and parameter at their estimated values. See Additional file 5, 6, 7, 8, 9, 10 for details. Consistent with original publication of the data [20], our results showed that almost all DNA methylation alterations were in the direction of reduction when resistant cell lines were compared with wild type, suggesting a homogenous pattern of DNA methylation during the acquisition of drug resistance. Furthermore, the OHT and ICI cell lines shared similar yet holded unique association patterns. It is noted that a proportion of genes are hypomethylated with down regulation of gene expression, suggesting the involvement of other genetic and epigenetic factors in the regulation process.

Although there exists a weak correlation between DNA methylation at promoter regions and gene expression for the three cell lines studied, the correlation of methylation and gene expression alterations, when comparing OHT/ICI to WT at the global level, is essentially 0. This implies that the relation between alterations in DNA methylation at promoter region and gene expression is gene-specific and, likely due to the involvement of other factors.

## Competing interests

The authors declare that they have no competing interests.

## Authors' contributions

JJ and CS conceived and designed the statistical model, and drafted the manuscript. JJ implemented the algorithm and carried out simulation studies and real data analysis. LL and YL participated in algorithm and manuscript revision. KPN and THH generated the experimental data, provided biological input and revised the manuscript. All authors read and approved the final manuscript.

## Pre-publication history

The pre-publication history for this paper can be accessed here:

http://www.biomedcentral.com/1755-8794/3/55/prepub

## Supplementary Material

Additional file 1 **Table S1 -- Gene expression data structure**. This table shows the gene expression data structure in both group: wild type and antiestrogen resistant group. Click here for file

Additional file 2 **Table S2 -- Gene lists which are hypomethylated and up-regulated**. This table presents gene lists obtained by using three different cutoff values with the OHT data set. Click here for file

Additional file 3 **Figure S1 -- Venn diagram presenting gene overlap**. Each figure is obtained using three different cutoff values. Based on each cutoff values, the status of each gene is determined in both data sets. The number of gene overlaps which were obtained by using gene expression data in both data set is calculated. The numbers in each Venn diagram presents the number of common genes in both data sets. Click here for file

Additional file 4 **Figure S2 -- Histogram of residuals**. This histogram is based on standardized residuals obtained by using estimates in our model; Top: residuals histogram of gene expression in ICI; Bottom: residuals histogram of methylation in ICI. Click here for file

Additional file 5 **Figure S3 -- Q-Q plot of residuals**. Each Q-Q plot is based on standardized residuals obtained by using parameter estimates in our model in ICI; Left: this plot is obtained by using gene expression residuals; Right: this plot is obtained by using methylation residuals. Click here for file

Additional file 6 **Figure S4 -- Histogram of gene effect**. Each histogram is based on estimated gene effect in our model in ICI; Top: these plots are obtained by using estimated gene effect of each group in gene expression (Left:WT and Right:ICI); Bottom: these plots are obtained by using estimated gene effect of each group in methylation (Left:WT and Right:ICI). Click here for file

Additional file 7 **Figure S5 -- Q-Q plot of gene effect**. Each Q-Q plot is based on estimated gene effect in our model in ICI; Top: these plots are obtained by using estimated gene effect of each group in gene expression (Left:WT and Right:ICI); Bottom: these plots are obtained by using estimated gene effect of each group in methylation (Left:WT and Right:ICI). Click here for file

Additional file 8 **Figure S6 -- Histogram of added probe effect**. Each histogram is based on estimated probe effect in our model in ICI; Left: these plots are obtained by using estimated added probe effect in gene expression; Right: these plots are obtained by using estimated added probe effect in methylation. Click here for file

Additional file 9 **Figure S7 -- Q-Q plot of added probe effect**. Each Q-Q plot is based on estimated probe effect in our model in ICI; Left: these plots are obtained by using estimated added probe effect in gene expression; Right: these plots are obtained by using estimated added probe effect in methylation. Click here for file

Additional file 10 **Supplementary materials -- Details about modeling and estimation**. This file includes details about marginal modeling and parameter estimation. Also, the exact form of parameter estimators are given. Click here for file
