# Supplementary material for: An empirical Bayes model for gene expression and methylation profiles in antiestrogen resistant breast cancer
Source: BMC Med Genomics. 2010 Nov 25;3:55. doi: 10.1186/1755-8794-3-55 (PMC3003621; doi:10.1186/1755-8794-3-55)
Supplement: Additional file 1 — Table S1 -- Gene expression data structure. This table shows the gene expression data structure in both group: wild type and antiestrogen resistant group. [file 1755-8794-3-55-S1.PDF]

Table S1: The data structure of gene expression

		group 1				group 2			
		k=1	k=2	k=3	k=4	k=1	k=2	k=3	k=4
Gene 1	b_{11}	G_{1111}	G_{1121}	G_{1131}	G_{1141}	G_{1112}	G_{1122}	G_{1132}	G_{1142}
	b_{12}	G_{1211}	G_{1221}	G_{1231}	G_{1241}	G_{1212}	G_{1222}	G_{1232}	G_{1242}
	$\vdots$	$\vdots$	$\vdots$	$\vdots$	$\vdots$	$\vdots$	$\vdots$	$\vdots$	$\vdots$
	b_{1J_1}	G_{1J_111}	G_{1J_121}	G_{1J_131}	G_{1J_141}	G_{1J_112}	G_{1J_122}	G_{1J_132}	G_{1J_142}
$\vdots$	$\vdots$	$\vdots$	$\vdots$	$\vdots$	$\vdots$	$\vdots$	$\vdots$	$\vdots$	$\vdots$
Gene I	b_{I1}	G_{I111}	G_{I121}	G_{I131}	G_{I141}	G_{I112}	G_{I122}	G_{I132}	G_{I142}
	b_{I2}	G_{I211}	G_{I221}	G_{I231}	G_{I241}	G_{I212}	G_{I222}	G_{I232}	G_{I242}
	$\vdots$	$\vdots$	$\vdots$	$\vdots$	$\vdots$	$\vdots$	$\vdots$	$\vdots$	$\vdots$
	b_{IJ_I}	G_{IJ_I11}	G_{IJ_I21}	G_{IJ_I31}	G_{IJ_I41}	G_{IJ_I12}	G_{IJ_I22}	G_{IJ_I32}	G_{IJ_I42}
