# Supplementary material for: An empirical Bayes model for gene expression and methylation profiles in antiestrogen resistant breast cancer
Source: BMC Med Genomics. 2010 Nov 25;3:55. doi: 10.1186/1755-8794-3-55 (PMC3003621; doi:10.1186/1755-8794-3-55)
Supplement: Additional file 2 — Table S2 -- Gene lists which are hypomethylated and up-regulated. This table presents gene lists obtained by using three different cutoff values with the OHT data set. [file 1755-8794-3-55-S2.PDF]

1 Statistical modeling

We merged gene expression and methylation status data together. First of all, we model on marginal data each and then we consider joint model on merged data.

1.1 Modeling on Gene Expression

We have two groups having the same data structure which can be used to compare each other: wild type(WT) and resistant group. Additionally, we have two different resistant groups: hydroxytamoxifen(OHT) resistant group and fulvestrant(ICI) resistant group. Here we have two different data sets obtained from two different resistant cell lines: OHT resistant v.s WT and ICI resistant v.s. WT. In each group, there are four replicates. The model we use here is given:

$$G_{ijkl} = \mu_{il} + b_{ij} + \epsilon_{ijkl},\ i = 1, \cdots, I,\ j = 1, \cdots, J_i,\ k = 1, \cdots, 4,\ l = 1, 2$$

where μ_{il} is gene effect in each group, b_{ij} probe effect in each gene and ϵ_{ijkl} is error term. We assume normality and each component in the model has normal distributions:

$$b_{ij} \sim N(0, \sigma^2), \quad \epsilon_{ijkl} \sim N(0, \delta^2)$$

$$(\mu_{i1}, \mu_{i2})^t \sim N((\mu_1, \mu_2)^t, \Sigma_1)$$

The following linear model shows us more details about the experimental design and notations in our model. For specific gene i, we have a linear model as follows:

$$G_i = \triangle_{1i}\beta_{1i} + \epsilon_{1i}$$

where

$$\triangle_{1i} = \left[\begin{array}{cc|cccc} 1_4 & 0 & 1_4 & & & \\ \vdots & \vdots & & \ddots & & \\ 1_4 & 0 & & & & 1_4 \\ \hline 0 & 1_4 & 1_4 & & & \\ \vdots & \vdots & & \ddots & & \\ 0 & 1_4 & & & & 1_4 \end{array}\right], \quad \beta_{1i} = \begin{pmatrix} \mu_1 \\ \mu_2 \\ b_1 \\ \vdots \\ b_{J_i} \end{pmatrix}, \text{ and}$$

$$G_i = (G_{i111}, G_{i121}, G_{i131}, G_{i141}, G_{i211}, G_{i221}, G_{i231}, G_{i241}, \cdots, G_{iJ_i11}, \cdots, G_{iJ_i41}, \cdots)^t.$$

The distribution assumptions for each component in our model can be represented as:

$$\beta_{1i} \sim N({\mu_{1i}}^*, {\Sigma_{1i}}^*), \quad {\mu_1}^* = (\mu_1, \mu_2, {0_{p1i}}^t)^t,\ {\Sigma_{1i}}^* = \begin{pmatrix} \Sigma_1 & 0 \\ 0 & \sigma^2 I_{p1i} \end{pmatrix}$$

and

$$\epsilon_{1i} \sim N(0, \delta^2 I_{gi}).$$

Notice that $p1i$ is the number of probes in gene i and $gi = 8 \times p1i$. Thus, the gene expression of gene i in the our linear model has the following distribution:

$$G_{i|\beta_{1i}} \sim N(\triangle_{1i}\beta_{1i},\ \delta^2 I_{gi}).$$

1.2 Modeling on Methylation

The data structure in methylation is exactly the same as the situations in gene expression except replicate. i.e., there are two groups, but no replicate, which is the only difference between them. The model we use here is given:

$$M_{ihl} = \eta_{il} + a_{ih} + d_{ihl},\ i = 1, \cdots, I,\ h = 1, \cdots, H_i,\ l = 1, 2$$

where η_{il} is gene effect in each group, a_{ih} probe effect in each gene, and d_{ihl} error terms. Again, we assume normality condition and each component in the model above has the following distributions:

$$a_{ih} \sim N(0, \omega^2),\ d_{ihl} \sim N(0, \tau^2)$$

$$(\eta_{i1}, \eta_{i2})^t \sim N((\eta_1, \eta_2)^t, \Sigma_2)$$

Thus, the linear model in the methylation data can be reexpressed as:

$$M_i = \triangle_{2i}\beta_{2i} + d_i$$

where

$$\triangle_{2i} = \left[\begin{array}{cc|ccc} 1 & 0 & 1 & & \\ \vdots & \vdots & & \ddots & \\ 1 & 0 & & & 1 \\ \hline 0 & 1 & 1 & & \\ \vdots & \vdots & & \ddots & \\ 0 & 1 & & & 1 \end{array}\right], \quad \beta_{2i} = \begin{pmatrix} \eta_{i1} \\ \eta_{i2} \\ a_1 \\ \vdots \\ a_{J_h} \end{pmatrix}$$

and

$$M_i = (M_{i11}, M_{i21}, M_{i31}, M_{i41}, M_{i12}, M_{i22}, M_{i32}, M_{i42}, M_{iJ_h1}, \cdots, M_{iJ_h2})^t.$$

Given the model above, the distributions of each component in our model follow:

$$\beta_{2i} \sim N({\eta_{1i}}^*, {\Sigma_{2i}}^*),\quad {\eta_{1i}}^* = (\eta_1, \eta_2, {0_{p2i}}^t)^t,\ {\Sigma_{2i}}^* = \begin{pmatrix} \Sigma_2 & 0 \\ 0 & \omega^2 I_{p2i} \end{pmatrix}$$

and

$$d_i \sim N(0, \tau^2 I_{mi}).$$

Notice that $p2i$ is the number of probes in gene i and $mi = 2 \times p2i$. Thus, the methylation of gene i in our linear model has the following distribution:

$$M_{i|\beta_{2i}} \sim N(\triangle_{2i}\beta_{2i},\ \tau^2 I_{mi}).$$

1.3 Co-integrated model

Here we consider modeling on merged data set, i.e., $D_i = ({G_i}^t, {M_i}^t)^t$. For specific gene i, the linear model for co-integrated data we use here is:

$$D_i = \triangle_i \beta_i + \epsilon_i$$

where

$$\triangle_i = \left[\begin{array}{cccc|ccccc}
1_4 & & & & 1_4 & & & & \\
\vdots & & & & & \ddots & & & \\
1_4 & & & & & & 1_4 & & \\
 & 1_4 & & & 1_4 & & & & \\
 & \vdots & & & & \ddots & & & \\
 & 1_4 & & & & & 1_4 & & \\ \hline
 & & 1 & & & & 1 & & \\
 & & \vdots & 0 & & & & \ddots & \\
 & & 1 & & & & & & 1 \\
 & & & 1 & & & 1 & & \\
 & & 0 & \vdots & & & & \ddots & \\
 & & & 1 & & & & & 1
\end{array}\right], \quad \beta_i = \begin{pmatrix} \mu_{i1} \\ \mu_{i2} \\ \eta_{i1} \\ \eta_{i2} \\ b_1 \\ \vdots \\ b_{J_i} \\ a_1 \\ \vdots \\ a_{J_h} \end{pmatrix}.$$

The distribution assumption for each component in our model is given:

$$(\mu_{i1}, \mu_{i2}, \eta_{i1}, \eta_{i2})^t \sim N(\mu, \Sigma), \;\; \beta_i \sim N({\mu_i}^*, \Sigma_{pi})$$

where

$${\mu_i}^* = \begin{pmatrix} \mu \\ 0_i \end{pmatrix}, \; \Sigma_{pi} = \begin{pmatrix} \Sigma & 0 & 0 \\ 0 & \sigma^2 I_{p1i} & 0 \\ 0 & 0 & \omega^2 I_{p2i} \end{pmatrix}.$$

Note that $p1i$ and $p2i$ are the number of probes within gene i. Thus, the data, D_i has the following distribution:

$$D_{i|\beta_i} \sim N(\triangle_i \beta_i, \begin{pmatrix} \delta^2 I_{gi} & 0 \\ 0 & \tau^2 I_{mi} \end{pmatrix})$$

where $gi = 8 \times p1i$ and $mi = 2 \times p2i$. As we can see, our model has hierarchical structure below:

$$\beta_i \sim N({\mu_i}^*, \Sigma_{pi}),$$
$$D_{i|\beta_i} \sim N(\triangle_i \beta_i, diag(\delta^2 I_{gi}, \tau^2 I_{mi}))$$

2 Estimation using EM

The model in the previous section involves unobservable variables. In order to get maximum likelihood estimates in such situations, we usually introduce

Expectation-Maximization (EM) algorithm. Basically, EM algorithm consists of two steps: Expectation and Maximization. In the expectation step, we calculate the conditional expectation of unobservable variable, β_i given observed data and parameter estimates obtained in the previous step. Given the estimate for unobservable variable, we derive maximum likelihood estimator of each parameter in the maximization step. These two steps are being done repeatedly in each iteration and we consider enough iterations until the convergence of EM algorithm is attained. Here we briefly describe EM algorithm applied to our case. More details are given in the book by McLachlan and Krishnan. (2007), the paper by Dempster et al. (1977).

2.1 E-step

In this step, we get estimate of unobservable variable through conditional expectation. In our model, we have unobservable variable, β_i. For iteration k, we get conditional expectation of the variable given observed data and parameter estimates obtained in the previous step via posterior distribution:

$$\begin{aligned}
[\beta_i|D_i] &\propto [D_i|\beta_i][\beta_i] \\
&\propto \exp\{-\tfrac{1}{2}(D_i-\Delta_i\beta_i)^t{\Sigma_e}^{-1}(D_i-\Delta_i\beta_i)-\tfrac{1}{2}(\beta_i-{\mu_i}^*)^t{\Sigma_{pi}}^{-1}(\beta_i-{\mu_i}^*)\} \\
&\propto \exp\{-\tfrac{1}{2}{\beta_i}^t({\Delta_i}^t{\Sigma_e}^{-1}\Delta_i+{\Sigma_{pi}}^{-1})\beta_i+[{D_i}^t{\Sigma_e}^{-1}\Delta_i+{\mu_i}^*{\Sigma_{pi}}^{-1}]\beta_i\} \\
&\propto \exp\{-\tfrac{1}{2}(\beta_i-K_i)^t{K_i^*}^{-1}(\beta_i-K_i)\}
\end{aligned}$$

where $K_i = {K_i^*}^{-1}[{\Delta_i}^t{\Sigma_e}^{-1}D_i+{\Sigma_{pi}}^{-1}{\mu_i}^*]$.

Thus,

$$Cov(\beta_i|D_i,\theta^{(k-1)}) = {\Delta_i}^t\hat{\Sigma}_e^{-1}\Delta_i+\hat{\Sigma}_{pi}^{-1} \equiv {V_i}^{(k)},$$

$$E(\beta_i|D_i,\theta^{(k-1)}) = {V_i}^{(k)}({\Delta_i}^t\hat{\Sigma}_e^{-1}D_i+\hat{\Sigma}_{pi}^{-1}\hat{\mu}_i^*) \equiv {\beta_i}^{(k)}.$$

Given the estimate of the unobservable variable, we proceed to the M-step.

2.2 M-step

After E-step, we try to get maximum likelihood estimator of parameters of interest by maximizing the following target function:

$$Q(\theta;\theta^{(k)}) \equiv E[\log L_c(\theta)|D_i,\theta^{(k)}]$$

where $\log L_c(\theta)$ is complete-data log likelihood function. The complete-data log likelihood is given:

$$l \equiv \log L_c(\theta) = \sum_{i=1}^{I}\{\log[D_i,\beta_i|\theta]\}$$

$$\propto \sum_{i=1}^{I}\{\log|\Sigma_e|+\log|\Sigma_{p_i}|+(D_i-\Delta_i\beta_i)^t{\Sigma_e}^{-1}(D_i-\Delta_i\beta_i)+(\beta_i-{\mu_i}^*)^t{\Sigma_{p_i}}^{-1}(\beta_i-{\mu_i}^*)\}$$

Since we assume normality, parameter estimates can be easily calculated with little algebra and the estimators of each parameter in our model have closed form, respectively. If we are interested in a parameter, to say μ, there are several constant terms with respect to the parameter in the complete-data log likelihood function, l. To make the function simple, corresponding constant terms with respect to each parameter are ignored in each situations. The brief formula derivation of all parameter estimators is given as follows:

•μ

$\frac{\partial l}{\partial \mu} = \frac{\partial}{\partial \mu}\{\sum_{i=1}^{I}(\beta_{1i}-\mu)^t\Sigma^{-1}(\beta_{1i}-\mu)\}$

$\therefore \mu^{(k)} = \frac{1}{I}\sum_{i=1}^{I}{\beta_{1i}}^{(k)}$

•δ^2

$\frac{\partial l}{\partial \delta^2} = \sum_{i=1}\{\frac{N_i}{\delta^2} - \frac{1}{(\delta^2)^2}{e_{1i}}^t e_{1i}\}$

$\therefore {\delta^2}^{(k)} = \frac{1}{N}\sum_{i=1}\{\mathrm{tr}(\Delta_{1i}{V_i}^{(k)}{\Delta_{1i}}^t) + {{e_{1i}}^{(k)}}^t {e_{1i}}^{(k)}\}$

•τ^2

$\frac{\partial l}{\partial \tau^2} = \sum_{i=1}\{\frac{M_i}{\tau^2} - \frac{1}{(\tau^2)^2}{e_{2i}}^t e_{2i}\}$

$\therefore {\tau^2}^{(k)} = \frac{1}{M}\sum_{i=1}\{\mathrm{tr}(\Delta_{2i}{V_i}^{(k)}{\Delta_{2i}}^t) + {{e_{2i}}^{(k)}}^t {e_{2i}}^{(k)}\}$

•Σ

$\frac{\partial l}{\partial \Sigma} = \sum_{i=1}\{\Sigma^{-1} - \Sigma^{-1}(\beta_{1i}-\mu)(\beta_{1i}-\mu)^t\Sigma^{-1}\}$

$\therefore \Sigma^{(k)} = \frac{1}{I}\sum_{i=1}\{{V_{1i}}^{(k)} + ({\beta_{1i}}^{(k)}-\mu)({\beta_{1i}}^{(k)}-\mu)^t\}$

•σ^2

$\frac{\partial l}{\partial \sigma^2} = \sum_{i=1}\{\frac{p_{1i}}{\sigma^2} - \frac{1}{(\sigma^2)^2}{\beta_{2i}}^t\beta_{2i}\}$

$\therefore {\sigma^2}^{(k)} = \frac{1}{\sum p_{1i}}\sum_{i=1}\{\mathrm{tr}({V_{2i}}^{(k)}) + {{\beta_{2i}}^{(k)}}^t {\beta_{2i}}^{(k)}\}$

•ω^2

$\frac{\partial l}{\partial \omega^2} = \sum_{i=1}\{\frac{p_{2i}}{\omega^2} - \frac{1}{(\omega^2)^2}{\beta_{3i}}^t\beta_{3i}\}$

$\therefore {\omega^2}^{(k)} = \frac{1}{\sum p_{2i}}\sum_{i=1}\{\mathrm{tr}({V_{3i}}^{(k)}) + {{\beta_{3i}}^{(k)}}^t {\beta_{3i}}^{(k)}\}$

where $p_{1i},\ p_{2i}$ are the number of probes of gene i in gene expression and methylation data set, respectively and $M = \sum p_{1i} \times 8,\ N = \sum p_{2i} \times 2$. Also,

$$ {V_i}^{(k)} = \left(\begin{array}{c|c|c} {V_{1i}}^{(k)} & & \\ \hline & {V_{2i}}^{(k)} & \\ \hline & & {V_{3i}}^{(k)} \end{array}\right) \text{ and } {\beta_i}^{(k)} = \begin{pmatrix} {\beta_{1i}}^{(k)} \\ {\beta_{2i}}^{(k)} \\ {\beta_{3i}}^{(k)} \end{pmatrix}. $$
