# Supplementary material for: An empirical Bayes model for gene expression and methylation profiles in antiestrogen resistant breast cancer
Source: BMC Med Genomics. 2010 Nov 25;3:55. doi: 10.1186/1755-8794-3-55 (PMC3003621; doi:10.1186/1755-8794-3-55)
Supplement: Additional file 3 — Figure S1 -- Venn diagram presenting gene overlap. Each figure is obtained using three different cutoff values. Based on each cutoff values, the status of each gene is determined in both data sets. The number of gene overlaps which were obtained by using gene expression data in both data set is calculated. The numbers in each Venn diagram presents the number of common genes in both data sets. [file 1755-8794-3-55-S3.PDF]

Table S2: Gene lists which are hypomethylated and up-regulated (OHT v.s. WT)

Criteria	Gene list
cutoff(0.7) (24 genes)	CPE,PHGDH,PPP2R5C,SLC20A1,LSS,TIMELESS,ENPP4,RGS10 SGCE,FADS1,RCOR3,RNF138,SLC27A6,ANLN,MYEF2,PSCD3,GTF2A1 C14orf106,FLJ14451,HECTD2,ZNF224,ZNF10,VIPR2,MDN1
cutoff(0.6) (46 genes)	TRA1,CPE,ETS2,PHGDH,MARCKS,PPP2R5C,SLC20A1,LSS,ACSL4 TIMELESS,TRIM14,TMPO,ENPP4,RGS10,CSPG2,SGCE,ATXN3,HMGCS1 SORBS1,CENPF,FADS1,MKI67,SLC7A1,KNS2,ADCY1,BRP44L,RCOR3 RNF138,C14orf10,SLC27A6,CHPT1,DDX50,RKHD1,ANLN,MYEF2,NSE1 PSCD3,GTF2A1,C14orf106,FLJ14451,HECTD2,CDAN1,ZNF224,ZNF10 VIPR2,MDN1
max value (137 genes)	TRA1,DPYSL2,SCD,CPE,SLC7A5,PKM2,ETS2,PHGDH,MARCKS PPP2R5C,SLC20A1,RBM6,BIRC5,PLK1,LSS,CYP51A1,FABP5 ACSL4,CYP1B1,HMGCR,WASPIP,CCNB2,SYNE2,UNC13B,TIMELESS SPTLC2,TRIM14,ABCC4,SLC25A12,PCF11,CCNA2,TMPO,SAT FANCA,ACTN2,ENPP4,RGS10,C2orf23,CSPG2,TROAP,SGCE ANAPC7,IARS,MLH3,PLK4,GPC4,MAP3K8,BARD1,ATXN3,HMGCS1 MICA,MICB,GAD1,XK,SORBS1,CENPF,EIF5,ATP1B3,FADS1 IRS2,KIAA1128,PIGH,EYA2,ABCG2,TRIP11,JAG2,GPR51,MTAP MKI67,PTOV1,FNBP4,SLC7A1,STAT5B,KIAA0582,KNS2,SYNJ1 ADCY1,ADAM22,ELYS,SF4,LUC7L2,BRP44L,RCOR3,MRPL39 FZD4,ZNF544,RNF138,C14orf10,MSRA,SLC27A6,MYLIP,MCM10 CENPJ,COLEC12,STX16,RRAGD,CHPT1,FLJ20364,DDX50,HEY1 RKHD1,ANLN,MYEF2,PRTFDC1,HDHD2,DLL1,NSE1,RAM2,PSCD3 RAPH1,GTF2A1,C6orf83,RNF157,C14orf106,LRRN1,FLJ14451 HECTD2,AP4E1,MGC15476,CDAN1,ZNF397,ZNF224,RNP,SYCP3 LOC387914,CENPH,EIF5A2,B4GALT6,ZNF10,GAS2L3,RPESP DLX1,VIPR2,ADAMTS19,LAMA3,ZNF519,MDN1
