# Supplementary figures and images for: An empirical Bayes model for gene expression and methylation profiles in antiestrogen resistant breast cancer
Source: BMC Med Genomics. 2010 Nov 25;3:55. doi: 10.1186/1755-8794-3-55 (PMC3003621; doi:10.1186/1755-8794-3-55)

### Maximum Probability

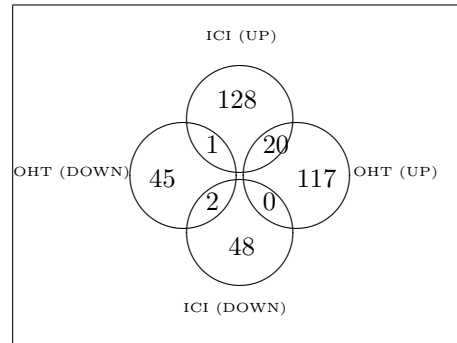

### Probability of 0.6

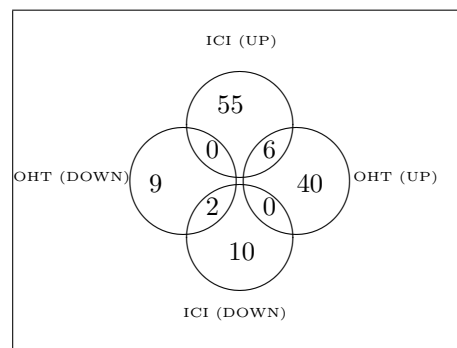

### Probability of 0.7

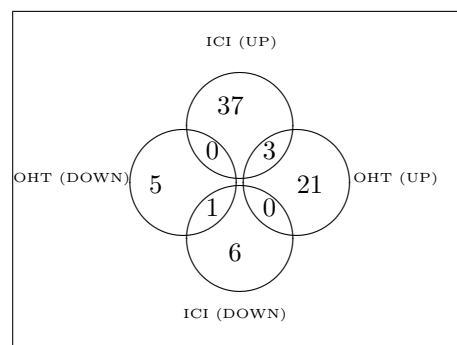

Supplement: Additional file 4 — Figure S2 -- Histogram of residuals. This histogram is based on standardized residuals obtained by using estimates in our model; Top: residuals histogram of gene expression in ICI; Bottom: residuals histogram of methylation in ICI. [file 1755-8794-3-55-S4.PDF]

residuals of GE in ICI

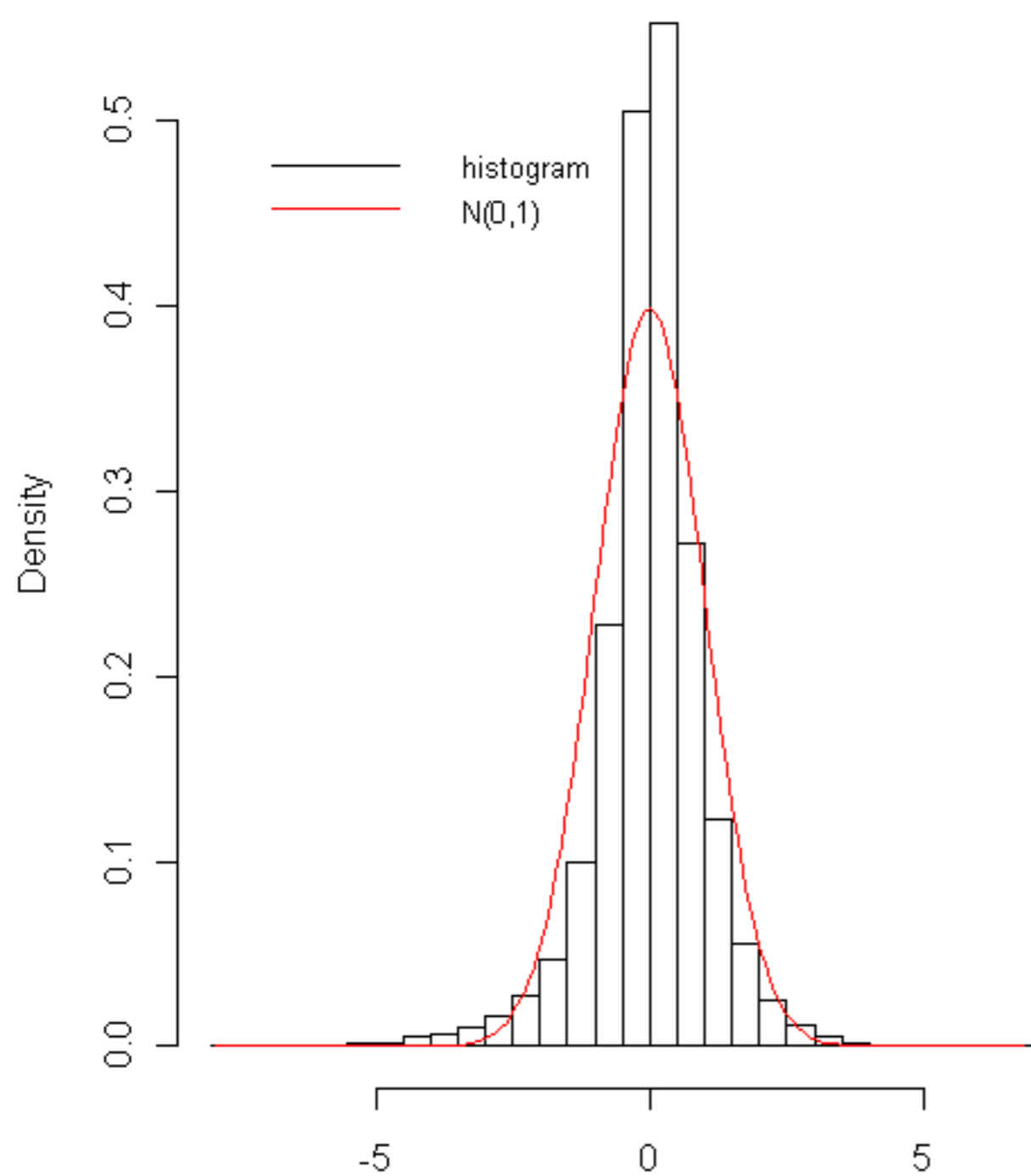

residuals of M in ICI

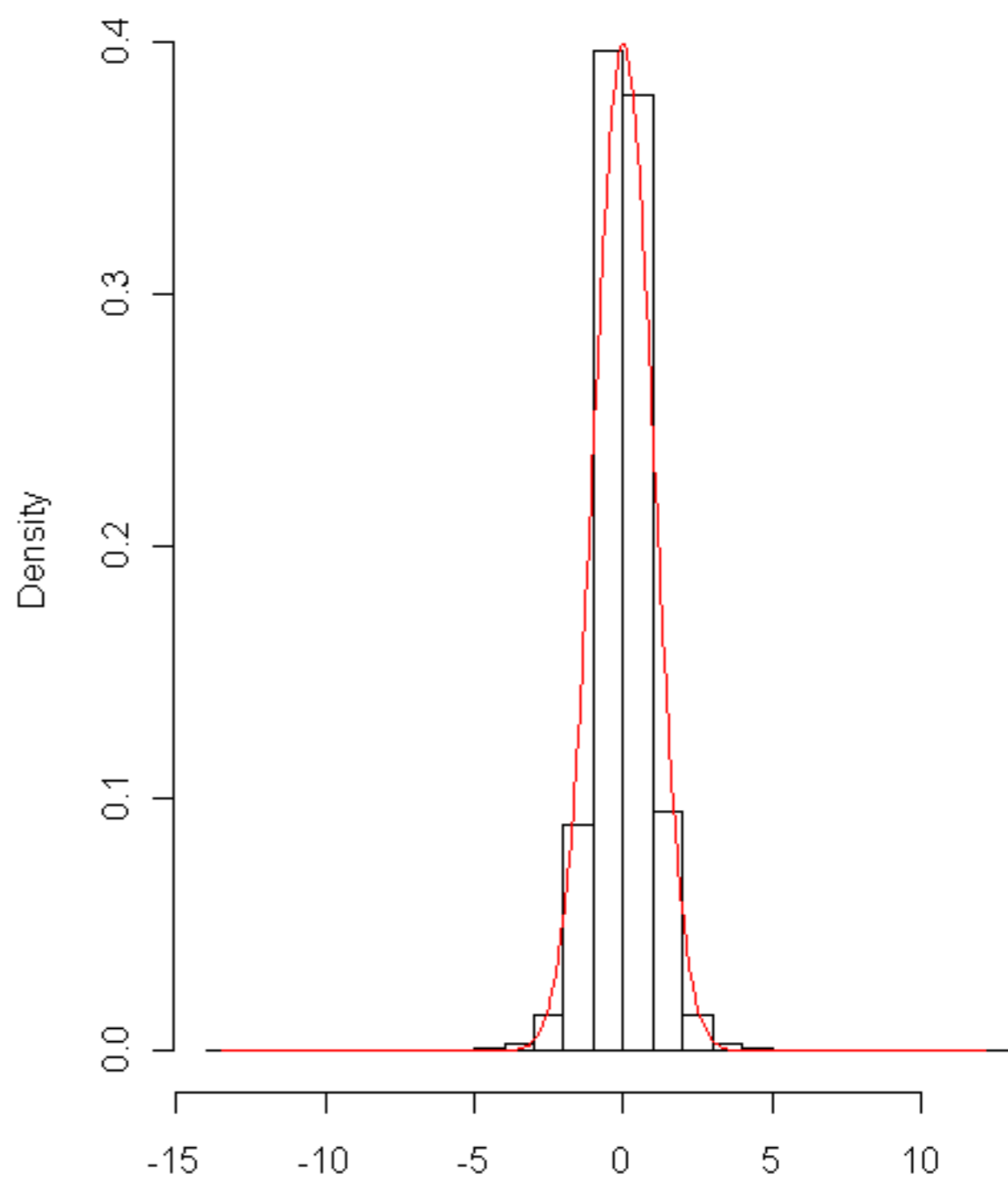

Supplement: Additional file 5 — Figure S3 -- Q-Q plot of residuals. Each Q-Q plot is based on standardized residuals obtained by using parameter estimates in our model in ICI; Left: this plot is obtained by using gene expression residuals; Right: this plot is obtained by using methylation residuals. [file 1755-8794-3-55-S5.PDF]

**Normal Q-Q Plot**

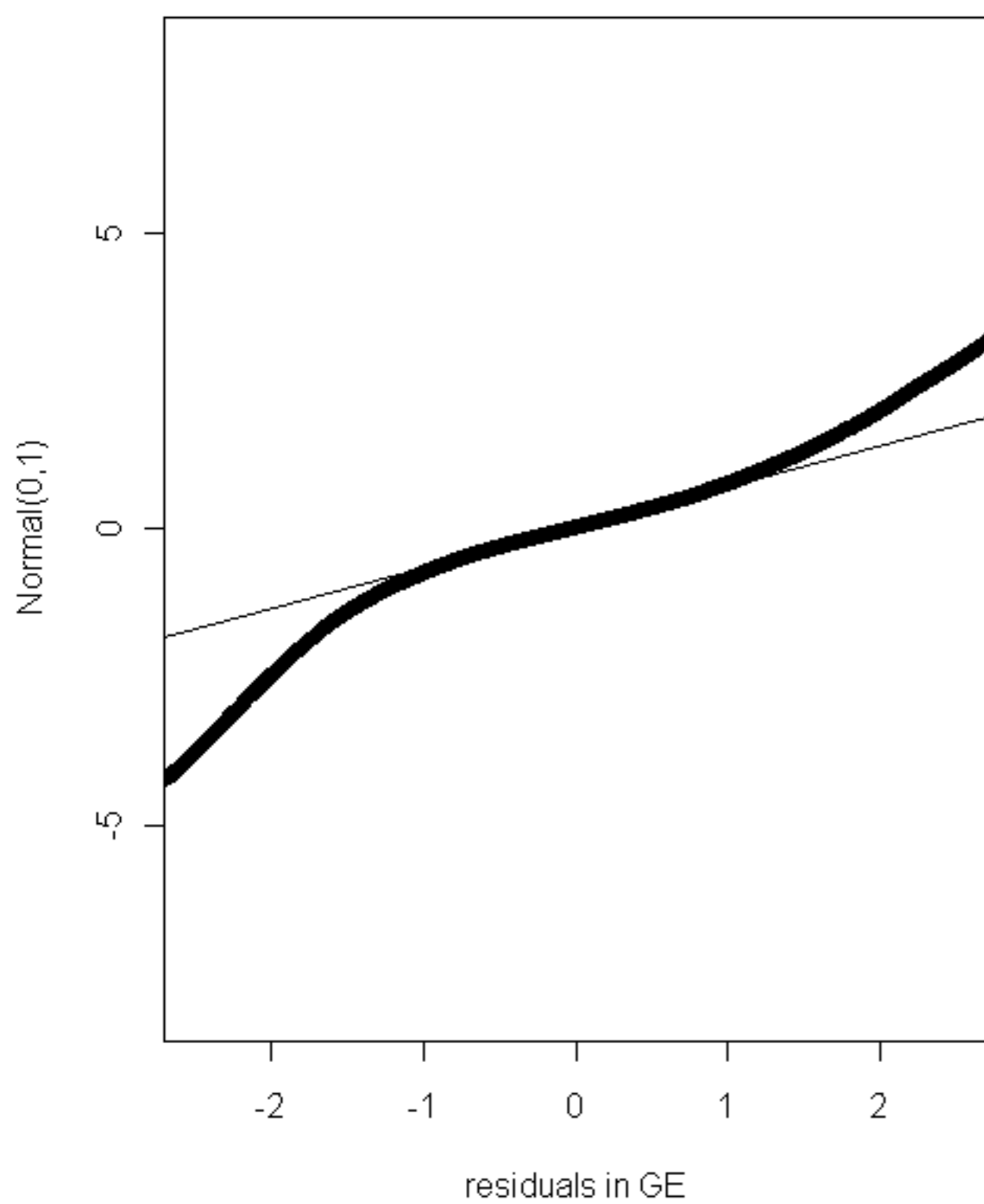

**Normal Q-Q Plot**

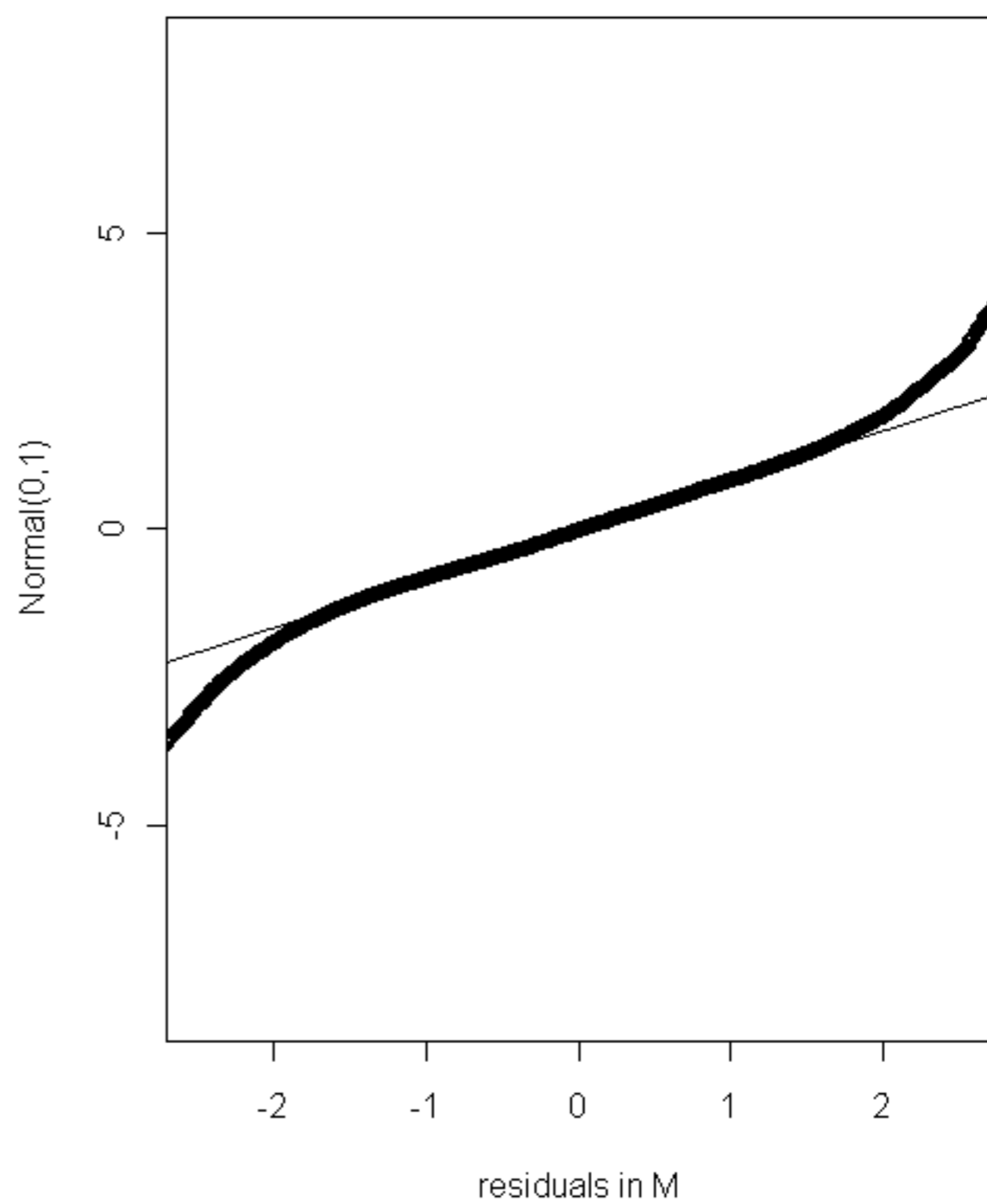

Supplement: Additional file 6 — Figure S4 -- Histogram of gene effect. Each histogram is based on estimated gene effect in our model in ICI; Top: these plots are obtained by using estimated gene effect of each group in gene expression (Left:WT and Right:ICI); Bottom: these plots are obtained by using estimated gene effect of each group in methylation (Left:WT and Right:ICI). [file 1755-8794-3-55-S6.PDF]

gene effects of WT in GE

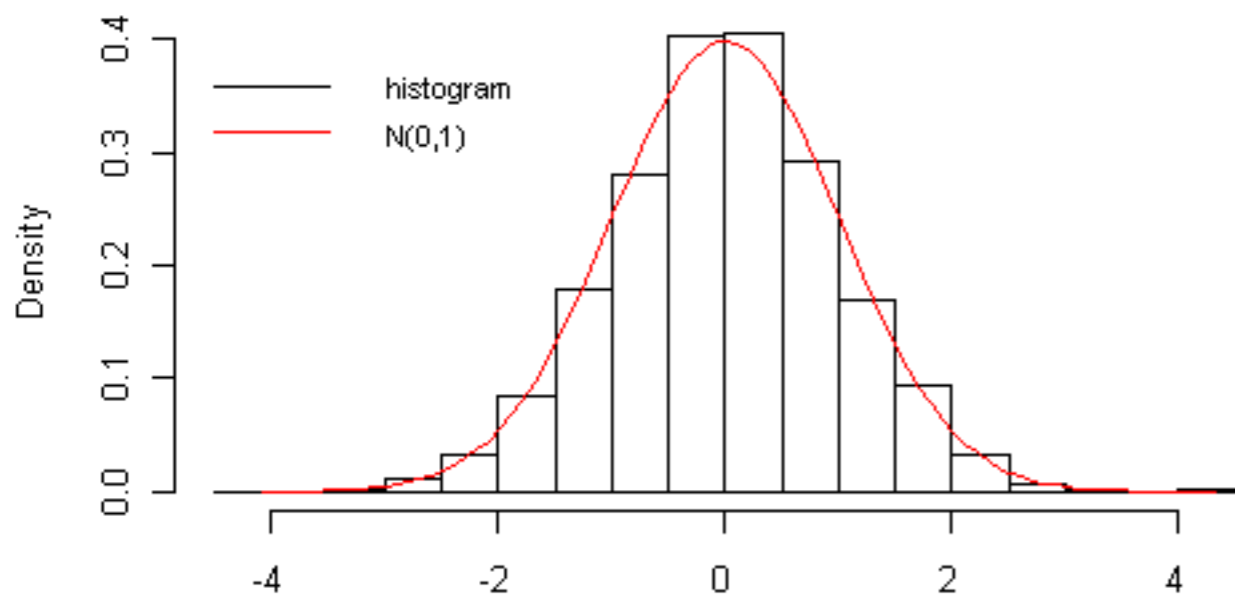

gene effects of ICI in GE

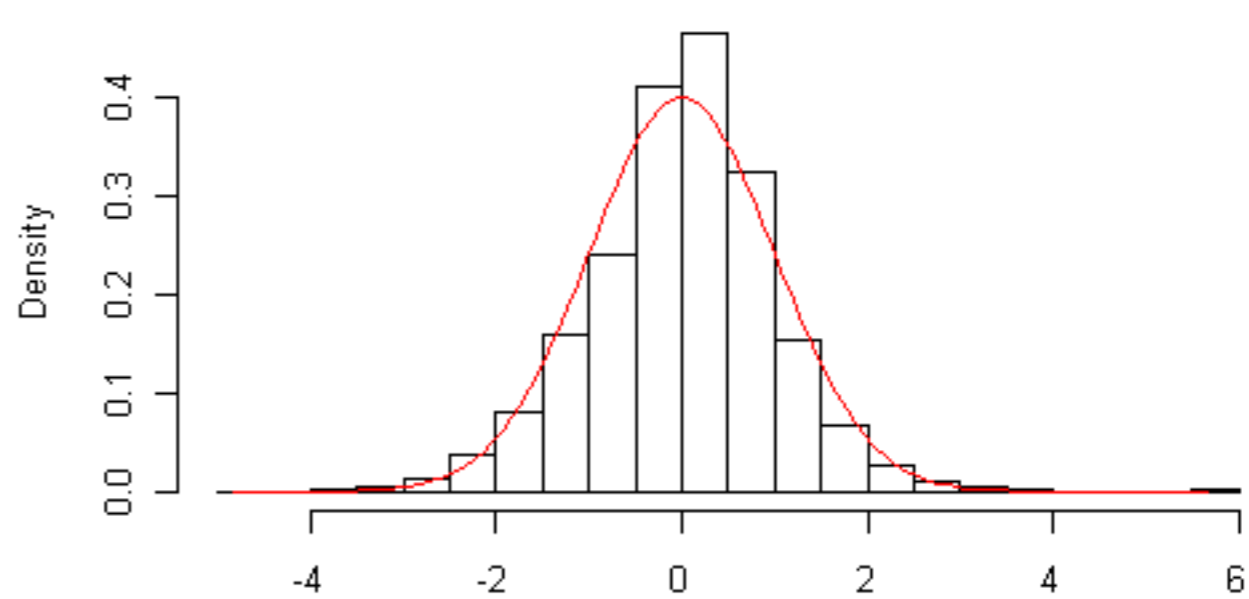

gene effects of WT in M

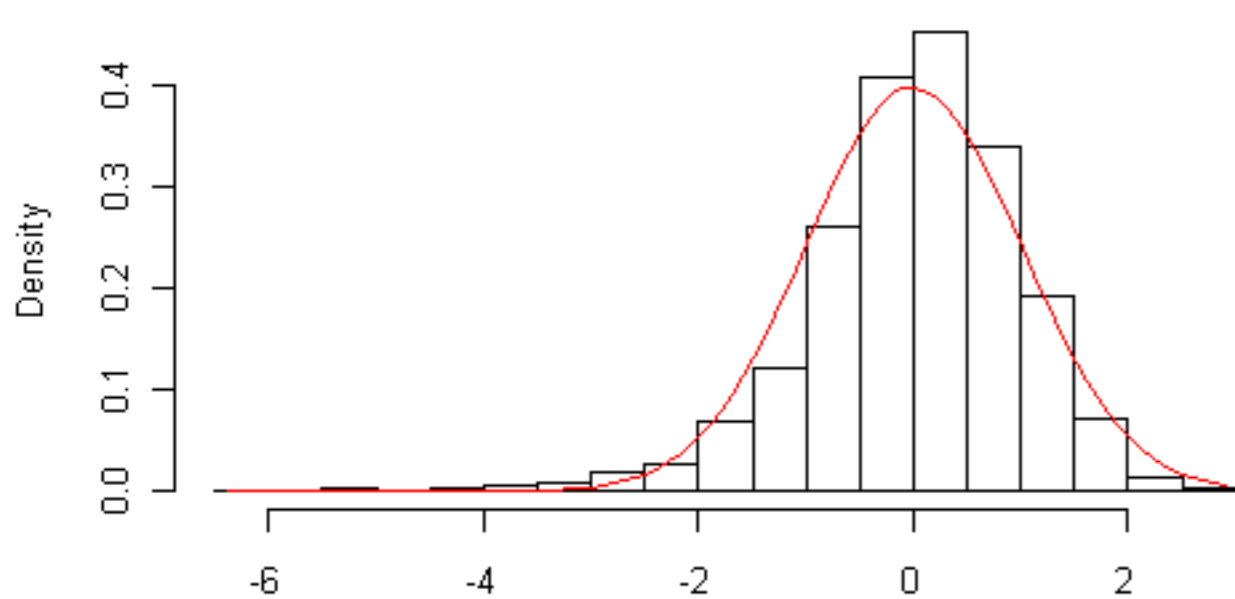

gene effects of ICI in M

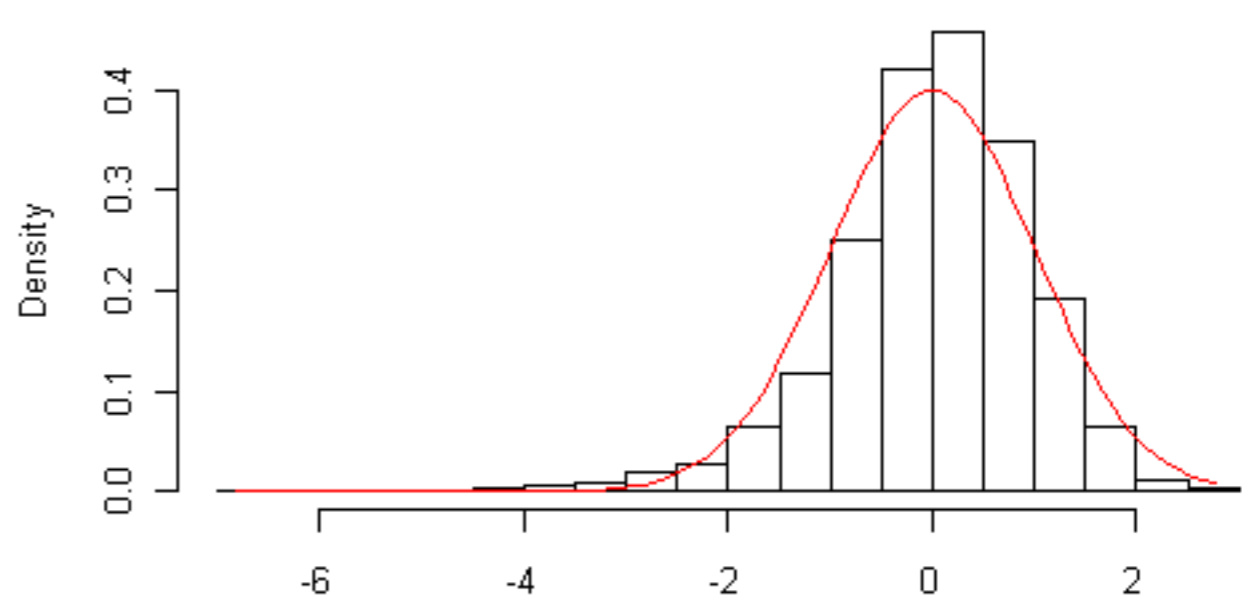

Supplement: Additional file 7 — Figure S5 -- Q-Q plot of gene effect. Each Q-Q plot is based on estimated gene effect in our model in ICI; Top: these plots are obtained by using estimated gene effect of each group in gene expression (Left:WT and Right:ICI); Bottom: these plots are obtained by using estimated gene effect of each group in methylation (Left:WT and Right:ICI). [file 1755-8794-3-55-S7.PDF]

WT gene effect in GE

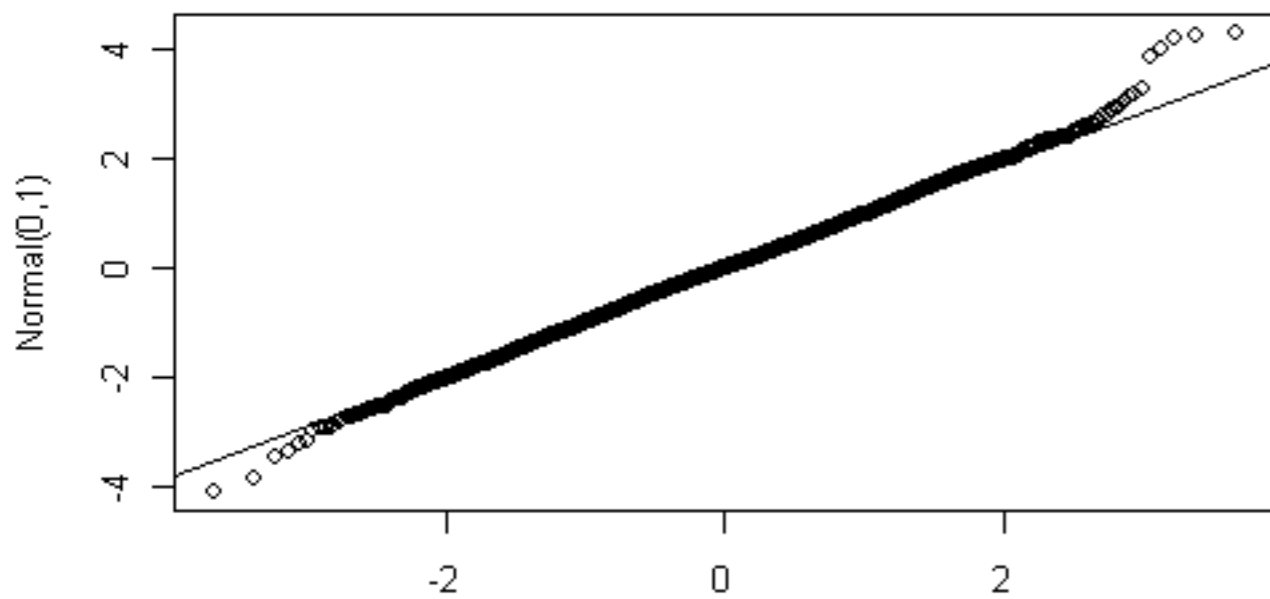

ICI gene effect in GE

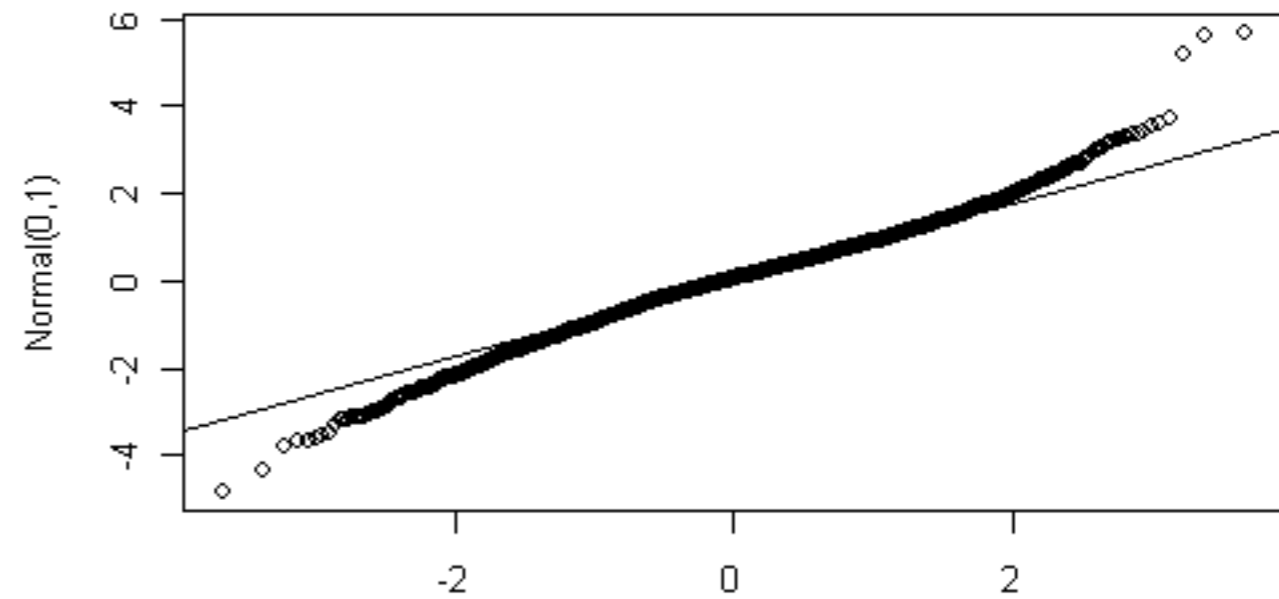

WT gene effect in M

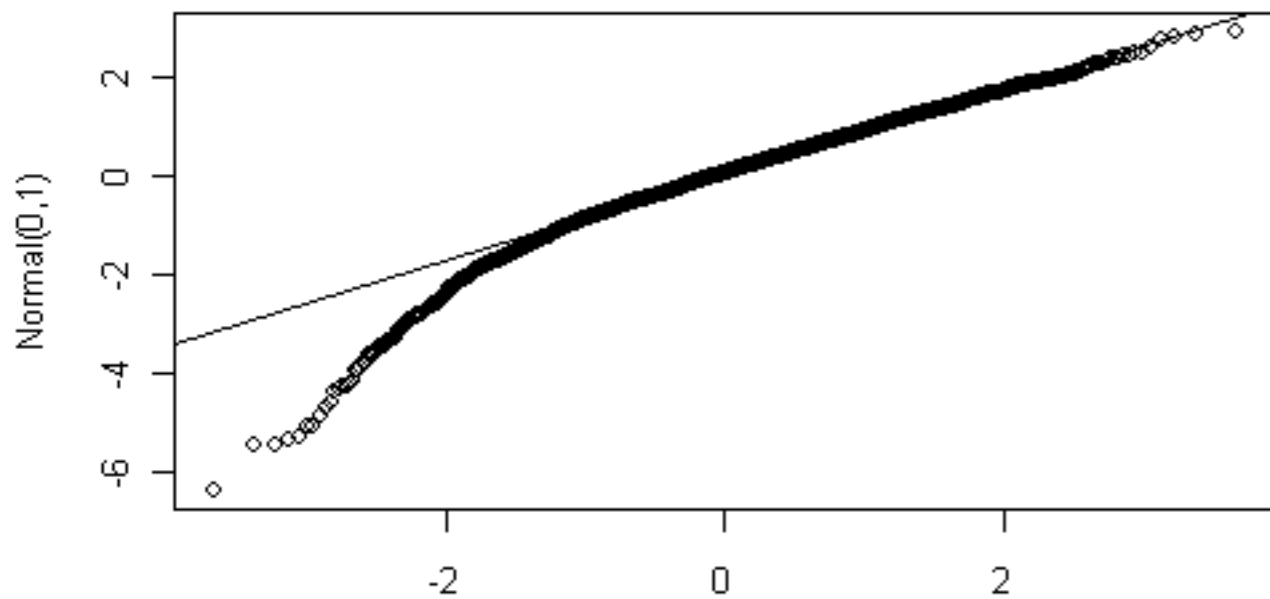

ICI gene effect in M

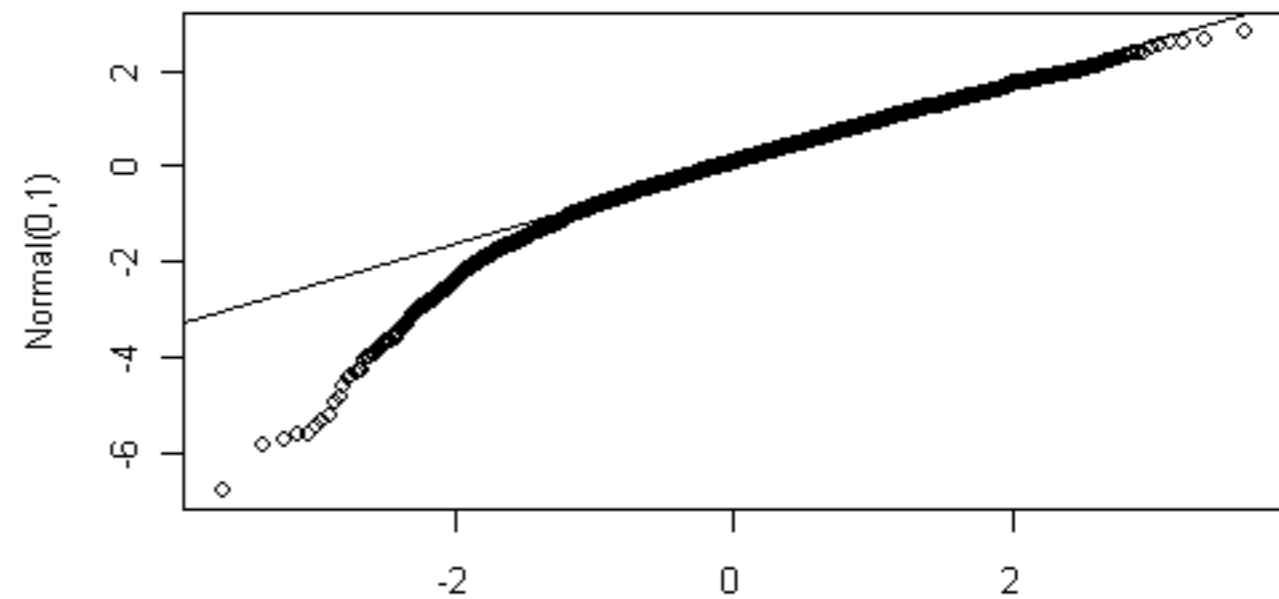

Supplement: Additional file 8 — Figure S6 -- Histogram of added probe effect. Each histogram is based on estimated probe effect in our model in ICI; Left: these plots are obtained by using estimated added probe effect in gene expression; Right: these plots are obtained by using estimated added probe effect in methylation. [file 1755-8794-3-55-S8.PDF]

**probe effects in GE**

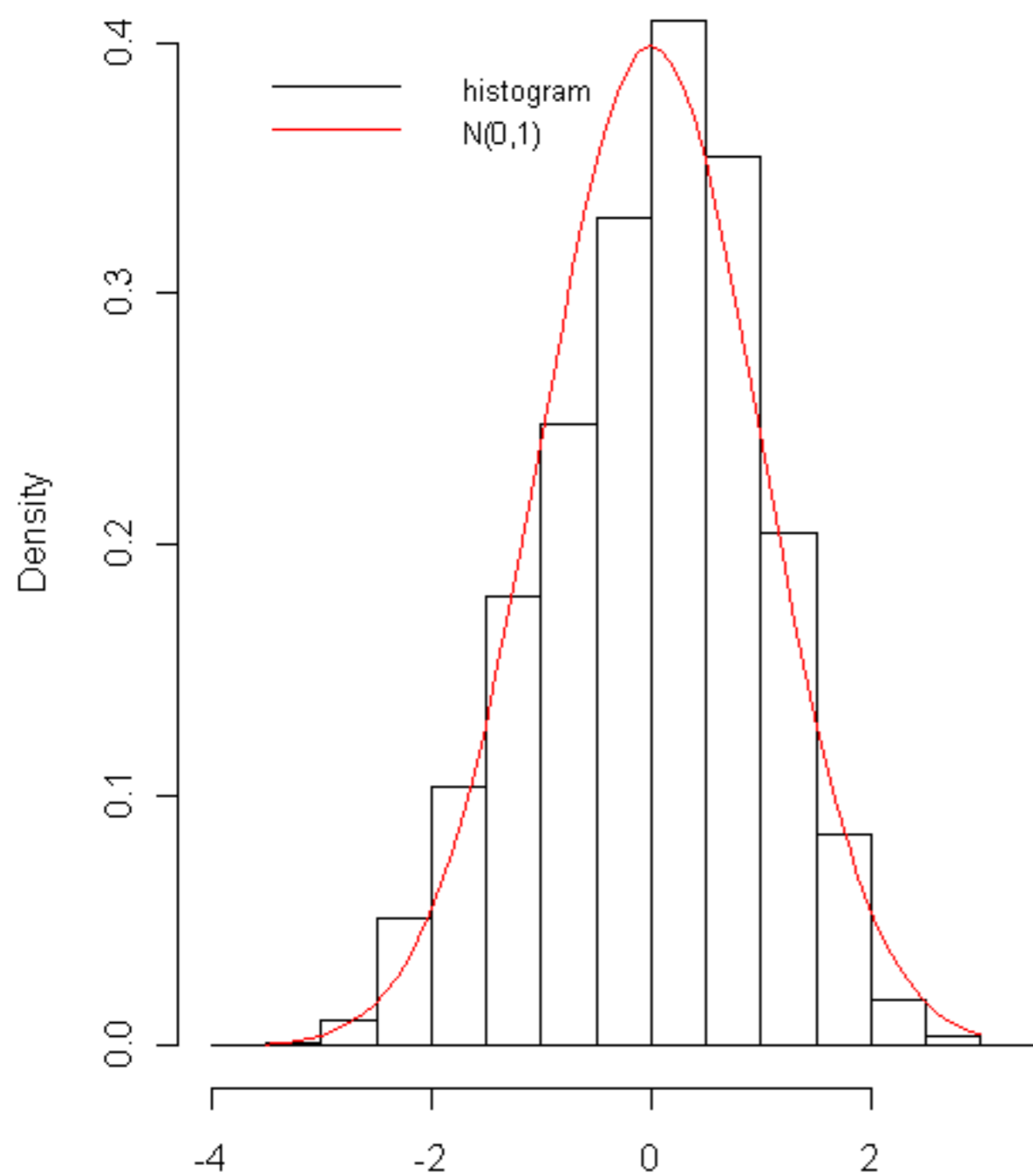

**probe effects in M**

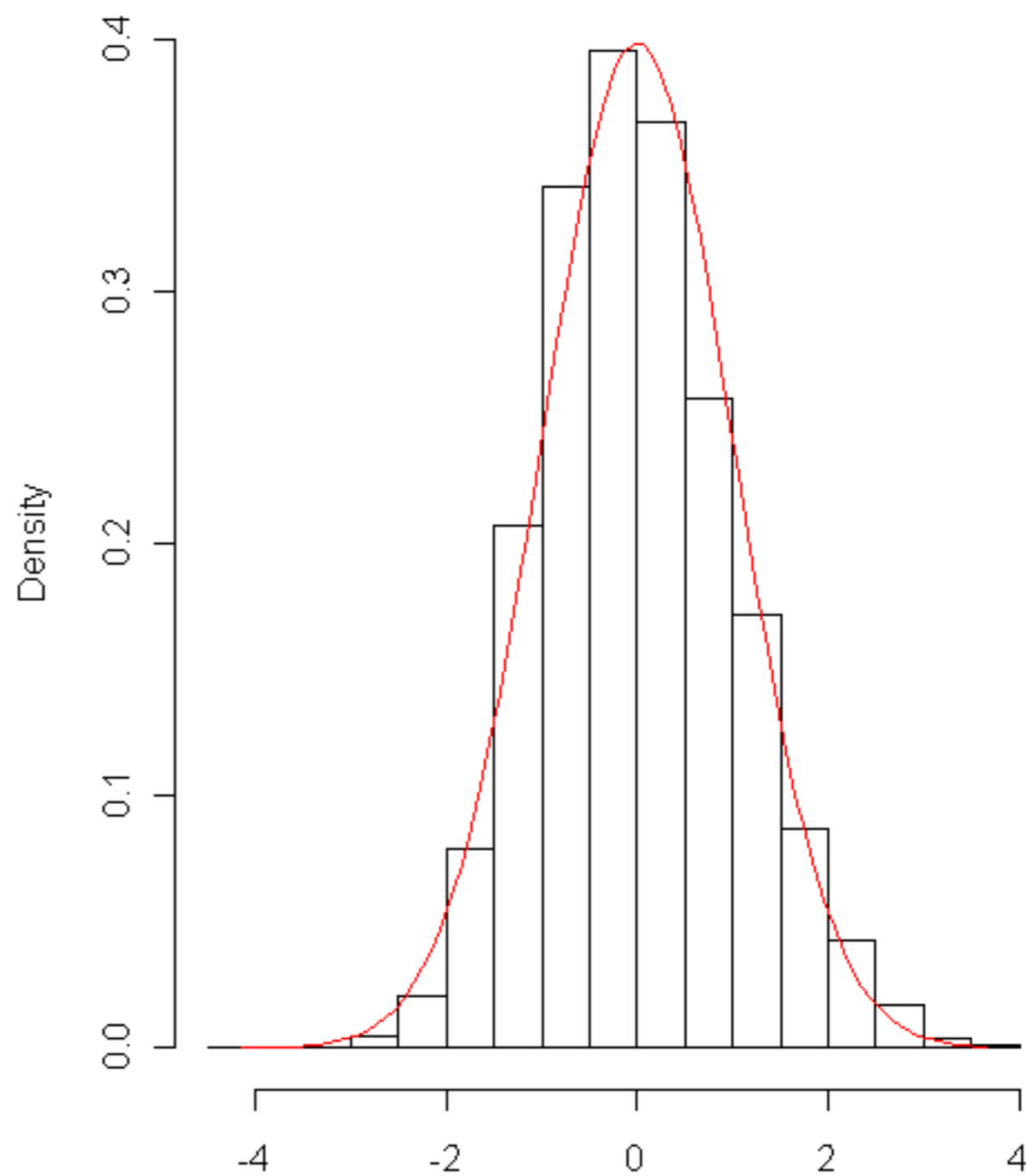

Supplement: Additional file 9 — Figure S7 -- Q-Q plot of added probe effect. Each Q-Q plot is based on estimated probe effect in our model in ICI; Left: these plots are obtained by using estimated added probe effect in gene expression; Right: these plots are obtained by using estimated added probe effect in methylation. [file 1755-8794-3-55-S9.PDF]

**probe effect in GE**

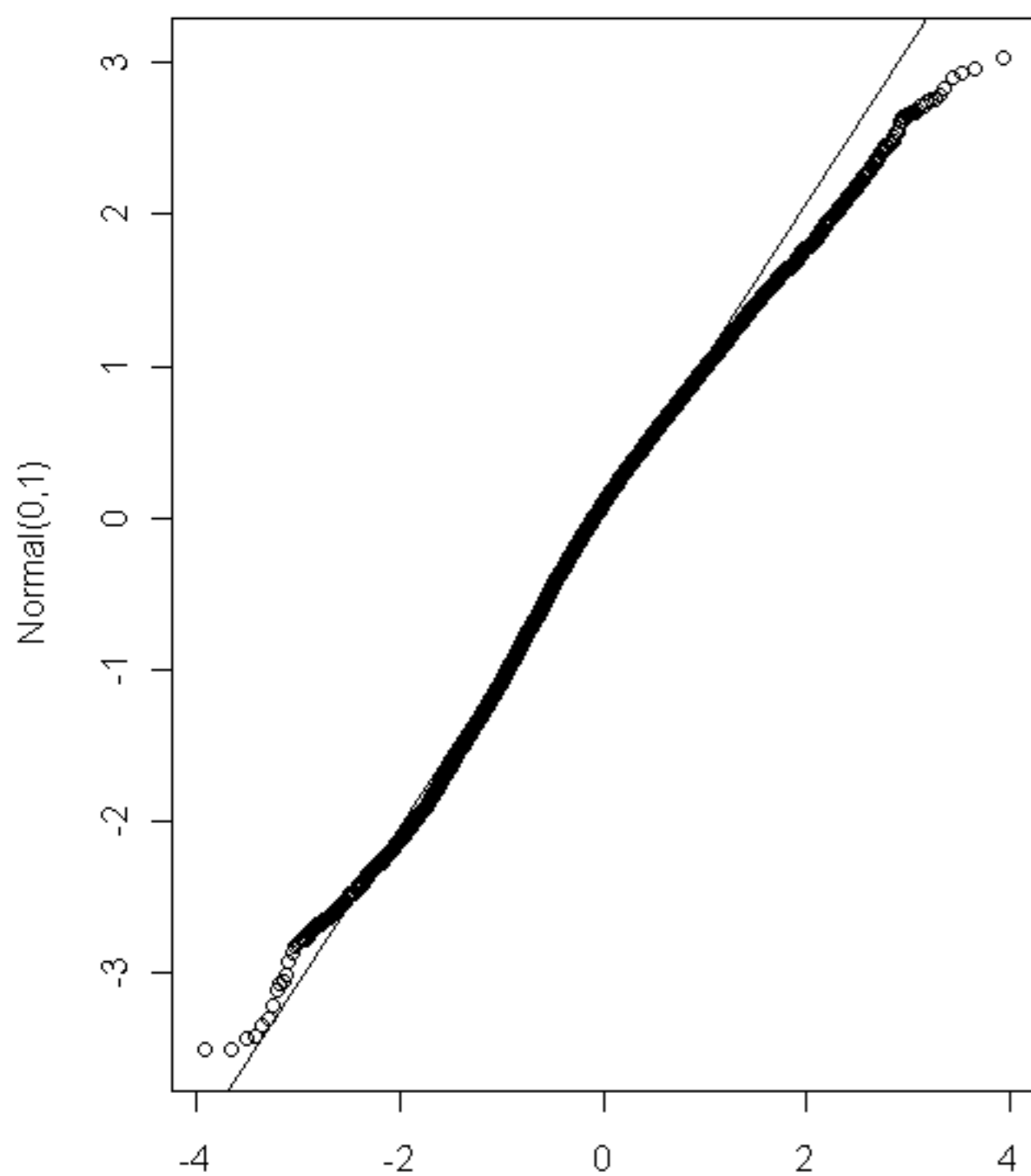

**probe effect in M**

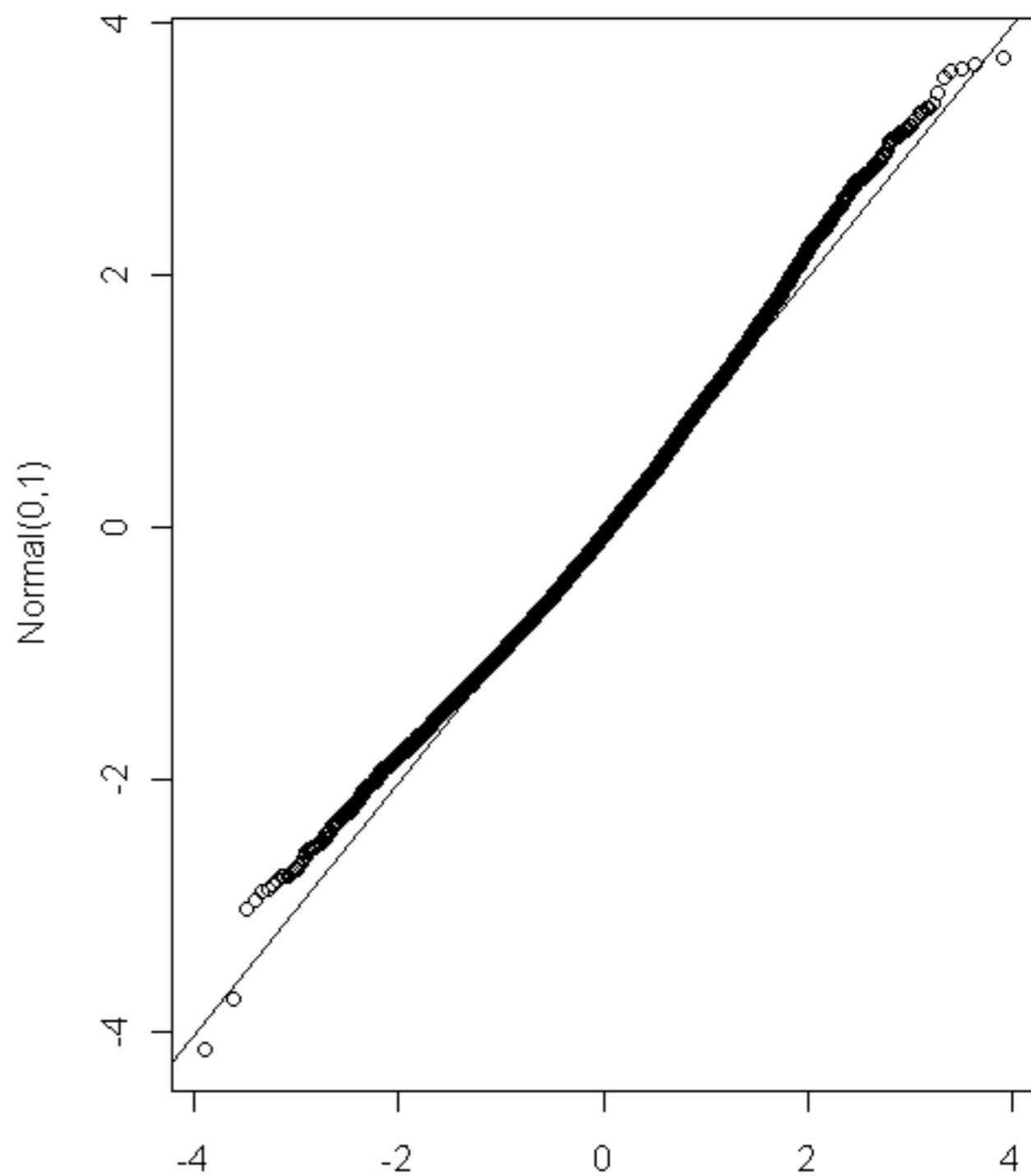

Supplement: Additional file 10 — Supplementary materials -- Details about modeling and estimation. This file includes details about marginal modeling and parameter estimation. Also, the exact form of parameter estimators are given. [file 1755-8794-3-55-S10.PDF]
